# The degradation of p53 and its major E3 ligase Mdm2 is differentially dependent on the proteasomal ubiquitin receptor S5a

**DOI:** 10.1038/onc.2013.413

**Published:** 2013-10-14

**Authors:** A Sparks, S Dayal, J Das, P Robertson, S Menendez, M K Saville

**Affiliations:** 1 Division of Cancer Research, Medical Research Institute, Jacqui Wood Cancer Centre, Ninewells Hospital and Medical School, University of Dundee, Dundee, UK; 2 Division of Molecular Medicine, College of Life Sciences, University of Dundee, Dundee, UK; 3 MRC Centre for Regenerative Medicine, University of Edinburgh, Edinburgh, UK

**Keywords:** p53, Mdm2, S5a, Rpn10, proteasome, proteasomal recognition

## Abstract

p53 and its major E3 ligase Mdm2 are both ubiquitinated and targeted to the proteasome for degradation. Despite the importance of this in regulating the p53 pathway, little is known about the mechanisms of proteasomal recognition of ubiquitinated p53 and Mdm2. In this study, we show that knockdown of the proteasomal ubiquitin receptor S5a/PSMD4/Rpn10 inhibits p53 protein degradation and results in the accumulation of ubiquitinated p53. Overexpression of a dominant-negative deletion of S5a lacking its ubiquitin-interacting motifs (UIM)s, but which can be incorporated into the proteasome, also causes the stabilization of p53. Furthermore, small-interferring RNA (siRNA) rescue experiments confirm that the UIMs of S5a are required for the maintenance of low p53 levels. These observations indicate that S5a participates in the recognition of ubiquitinated p53 by the proteasome. In contrast, targeting S5a has no effect on the rate of degradation of Mdm2, indicating that proteasomal recognition of Mdm2 can be mediated by an S5a-independent pathway. S5a knockdown results in an increase in the transcriptional activity of p53. The selective stabilization of p53 and not Mdm2 provides a mechanism for p53 activation. Depletion of S5a causes a p53-dependent decrease in cell proliferation, demonstrating that p53 can have a dominant role in the response to targeting S5a. This study provides evidence for alternative pathways of proteasomal recognition of p53 and Mdm2. Differences in recognition by the proteasome could provide a means to modulate the relative stability of p53 and Mdm2 in response to cellular signals. In addition, they could be exploited for p53-activating therapies. This work shows that the degradation of proteins by the proteasome can be selectively dependent on S5a in human cells, and that this selectivity can extend to an E3 ubiquitin ligase and its substrate.

## Introduction

The covalent conjugation of ubiquitin to proteins can target them for degradation by the 26S proteasome.^1^ This is composed of two multi-protein complexes:^2, 3, 4^ the 20S core and the 19S regulatory particle (RP). Protein degradation takes place in the central cavity of the 20S core. The 19S RP participates in the recruitment of ubiquitinated proteins. It also mediates protein deubiquitination, unfolding and translocation, and opens the gated entrance to the 20S core.

Ubiquitin-dependent recognition of substrates by the proteasome involves ubiquitin-binding receptors. Studies in lower organisms show that particular ubiquitin receptors participate in the recognition of specific subsets of ubiquitinated proteins.^5, 6^ Much remains to be learned regarding the identity and precise roles of proteasome-targeting ubiquitin receptors in man. The degree of substrate selectivity of ubiquitin receptors is unclear. In addition, the therapeutic potential of targeting ubiquitin-binding proteins has not been adequately explored. Bortezomib, an inhibitor of the proteolytic activity of the proteasome, is used in the treatment of some cancers.^7, 8^ Blocking proteasomal recruitment is an alternative mechanism for interfering with protein degradation by the proteasome.

There are over 20 families of ubiquitin-binding domains that are present in more than 200 proteins.^9^ These proteins are involved in mediating a number of ubiquitin-dependent processes, and it is unclear how many of them are involved in proteasomal recruitment. S5a/PSMD4/Rpn10 and ADRM1/Rpn13 are intrinsic subunits of the 19S RP that bind ubiquitin and are involved in substrate recognition.^10, 11, 12, 13^ There is evidence for additional ubiquitin-binding activity in the 19S RP.^14, 15^ A substantial proportion of S5a is extraproteasomal.^16, 17, 18, 19^ It remains to be established whether this participates in bringing ubiquitinated substrates to the proteasome. There are also other ubiquitin receptors that associate transiently with the proteasome in substoichiometric amounts. These receptors are thought to be involved in the shuttling of ubiquitinated proteins to the proteasome. A class of adaptor proteins contain a proteasome-binding ubiquitin-like (UBL) domain and a ubiquitin-binding ubiquitin-associated (UBA) domain.^11, 12, 13, 20^ Human S5a and ADRM1 interact with UBL domains in UBA/UBL proteins.^10, 21, 22^ Recruitment of proteins to the proteasome by S5a and ADRM1 could involve direct interaction with ubiquitinated substrates and/or indirect interactions bridged by adaptor proteins. Additional proteasome subunits may also interact with UBL domains,^12, 23^ and there are ubiquitin-binding proteins that associate with the proteasome independently of UBL domains.^24, 25, 26^

Mdm2 is a key repressor of p53.^27, 28^ It is an E3 ligase that promotes p53 ubiquitination and proteasomal degradation. In addition, binding of Mdm2 to p53 can directly inhibit the transcriptional activity of p53 through a number of mechanisms.^29, 30^ Mdm2 is ubiquitinated and is degraded by the proteasome.^31^ The proteasome consequently contributes to the maintenance of the balance between levels of p53 and Mdm2. This balance is critical for tumour suppression by p53 and has a considerable impact on the magnitude of p53 activation in response to cellular stresses.^32, 33, 34^ Inhibition of the proteasomal degradation of p53 and enhanced degradation of Mdm2 are involved in p53 activation in response to stress signals.^35, 36^ Interventions that stabilize both p53 and Mdm2, including inhibition of the proteolytic activity of the proteasome, can lead to the accumulation of sufficient Mdm2 to repress the transcriptional activity of p53.^30, 35, 37^

Despite the importance of the proteasome in the regulation of p53, the pathways through which ubiquitinated p53 and Mdm2 are recruited to the proteasome have not been defined. There is evidence that S5a influences p53 protein expression,^38, 39^ but this has not been investigated in detail. Consistent with a role in recruitment of p53 to the proteasome, knockdown of the UBA/UBL adaptor proteins hHR23A and hHR23B has been observed to cause the accumulation of p53.^40^ However, in other studies, their depletion resulted in a decrease in p53 levels.^41, 42, 43^ p53 and Mdm2 have been reported to bind to a number of proteasomal subunits in a ubiquitin-independent manner.^44, 45, 46, 47, 48^ Mdm2 promotes ubiquitination-independent association of p53 with the proteasome, but an additional step requiring the ubiquitin ligase activity of Mdm2 is necessary for p53 degradation.^46^

Our investigation of the regulation of p53 by the deubiquitinating enzyme USP5 raised the possibility that there are differences in proteasomal recognition of p53 and Mdm2.^49^ As a first step to explore this, we have looked at the role of the proteasomal ubiquitin receptor S5a. Targeting S5a inhibits p53 protein degradation without affecting the stability of Mdm2. This results in an increase in p53 expression and transcriptional activity. Our data are consistent with a key role of S5a in the recognition of p53 by the proteasome. It is possible that degradation of Mdm2 normally proceeds through an S5a-independent mechanism, or an alternative pathway of proteasomal recognition of Mdm2 may compensate for loss of S5a.

## Results

### S5a knockdown causes the accumulation of ubiquitinated p53

**Figure 1:**
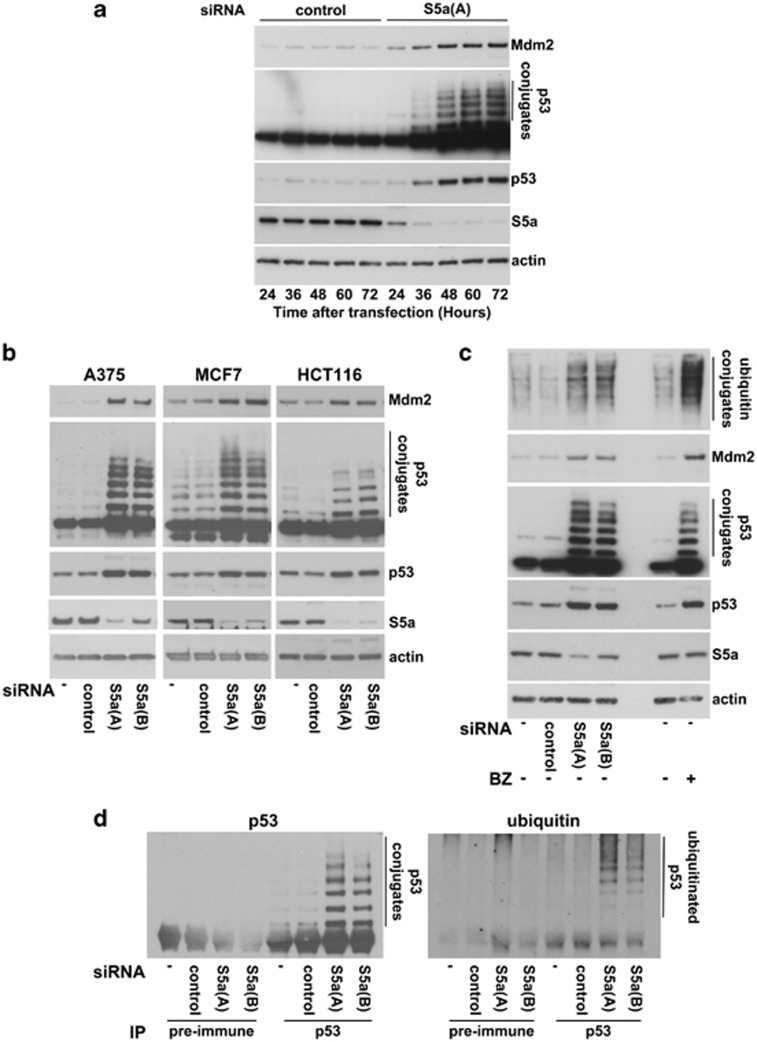
Depletion of S5a causes the accumulation of ubiquitinated p53. Cells were mock transfected (−) or transfected with non-targeting siRNA (control) or siRNA targeting S5a. siRNA S5a (A) and (B) are complementary to two different sequences in S5a. Unless otherwise indicated in these and subsequent experiments cells were harvested for western blotting 48 h after transfection under denaturing conditions to prevent protein deubiquitination. Where appropriate a short and an extended exposure of the western blot for p53 are presented in order to show total p53 levels and high-molecular weight p53 conjugates. (**a**) In A375 cells knockdown of S5a caused the accumulation of p53 and p53 conjugates with kinetics that paralleled the decrease in S5a. (**b**) Depletion of S5a increased the level of p53 and p53 conjugates in A375, MCF7 and HCT116 cells. (**c**) A375 cells were transfected as shown and, where indicated, the proteasome inhibitor bortezomib (BZ; 50 nM) was added 7 h before harvesting. These conditions are optimal for the effects of BZ on p53 levels and ubiquitination and on the general pattern of high-molecular weight ubiquitin conjugates. BZ and knockdown of S5a cause a similar increase in p53 and p53 conjugates. (**d**) Lysates of A375 cells were prepared under denaturing conditions to maintain protein ubiquitination and disrupt protein–protein interactions. Lysates were incubated with pre-immune rabbit serum or anti-p53 antibody CM.1, and immunoprecipitates were analysed by western blotting for p53 (left panel) or ubiquitin (right panel). High-molecular weight p53 conjugates detected after S5a knockdown contain ubiquitinated p53. A band corresponding to antibody heavy chain that co-migrates with unmodified p53 is presented in the western blots.

To investigate the pathway of proteasomal recognition of p53 and Mdm2, the ubiquitin receptor S5a was knocked down using synthetic siRNA. Blocking ubiquitin-dependent recognition of a substrate by the proteasome should result in an increase in the cellular level of the protein and in its ubiquitin-conjugated forms. Consistent with this, depletion of S5a resulted in accumulation of endogenous wild-type p53, and high-molecular weight p53 conjugates in A375 melanoma, MCF7 breast cancer and HCT116 colon cancer-derived cells (Figures 1a and b). S5a knockdown did not decrease the protein levels of other intrinsic proteasomal subunits (Supplementary Figure S1). In addition, as observed in lower organisms,^6, 50, 51, 52, 53, 54^ 26S proteasomes can be assembled that are depleted of S5a (Supplementary Figure S2). These data indicate that the effects on p53 are a specific consequence of S5a knockdown. Furthermore, activation of DNA damage pathways is not responsible for the upregulation of p53 following knockdown of S5a (Supplementary Figure S3).

S5a knockdown and bortezomib-mediated inhibition of the proteolytic activity of the proteasome caused similar increases in the level of p53 and high-molecular weight conjugates of p53 (Figure 1c). The effects of S5a depletion are thus consistent with inhibition of the proteasomal degradation of p53. Bortezomib caused greater accumulation of high-molecular weight ubiquitin conjugates than S5a knockdown. This is in line with studies in *Saccharomyces cerevisiae* which show that knockout of the yeast homologue of S5a affects the degradation of only a subset of ubiquitinated proteins targeted for proteasomal degradation.^5, 6^ Inhibitors of the proteolytic activity of the proteasome can deplete the pool of free ubiquitin.^55, 56, 57^ Indicative of a more limited effect on protein degradation S5a knockdown did not reduce free ubiquitin levels (Supplementary Figure S4).

Immunoprecipitation of p53 and western blotting with anti-ubiquitin antibodies confirmed that the high-molecular weight p53 conjugates accumulated after depletion of S5a contain ubiquitin (Figure 1d). The pattern of conjugates detected by p53 and anti-ubiquitin antibodies differed to some extent. Higher-molecular weight conjugates were more prominent in the ubiquitin western blots of the immunoprecipitated p53. This is likely to reflect the increasing ratio of ubiquitin to p53 epitopes in the higher-molecular weight conjugates. There may also be epitope masking of the ubiquitin molecules conjugated in the closest proximity to p53.

### Depletion of S5a selectively inhibits the degradation of p53

**Figure 2:**
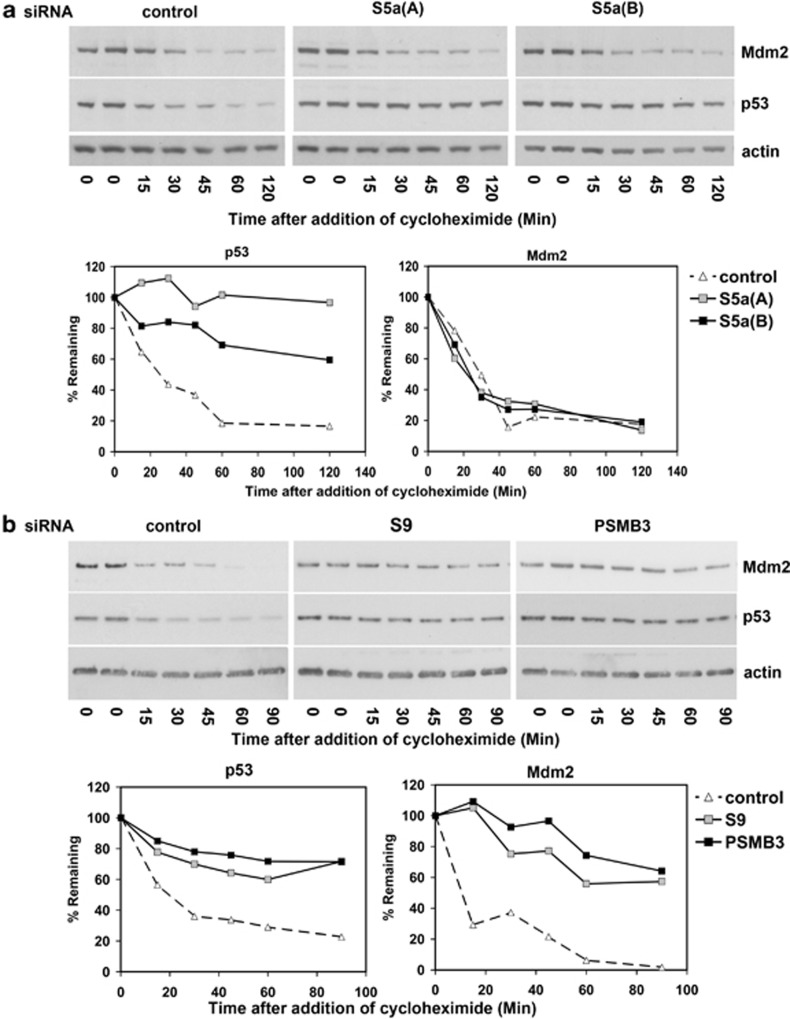
Knockdown of S5a selectively inhibits the degradation of p53 without effects on the degradation of Mdm2. A375 cells were transfected with non-targeting siRNA (control) or with siRNA targeting the indicated proteasomal subunits. Cells were treated with cycloheximide (20 μg/ml) for the indicated time before harvesting. Independent duplicate 0 min samples were analysed. The upper panels are western blots (different exposures are shown so that protein levels in the absence of cycloheximide are matched). The lower panels show quantification of the western blots. (**a**) S5a knockdown stabilizes p53 without affecting the stability of Mdm2. (**b**) Gross interference with the function of the 19S RP by knockdown of S9 or the 20S core of the proteasome by depletion of PSMB3 caused the stabilization of both p53 and Mdm2. The differential effect of S5a knockdown on the degradation of p53 and Mdm2 is selective in that it is not recapitulated by the depletion of other proteasomal subunits.

S5a knockdown inhibited p53 protein degradation while having no effect on the rate of Mdm2 degradation in all three cell lines examined (Figure 2a and Supplementary Figure S5). Mdm2 protein expression was elevated by S5a knockdown (Figure 1). *Mdm2* is a p53 target gene and we show below that this increase in Mdm2 levels is due to transcriptional activation of p53. Combined S5a knockdown and treatment with bortezomib also demonstrated that Mdm2 continues to be degraded by the proteasome in cells depleted of S5a (Supplementary Figure S6). This shows that Mdm2 is not degraded by a compensatory proteasome-independent pathway. Mdm2 is a key regulator of p53 stability in the cell lines used.^58, 59, 60^ Treatment of A375 cells with the specific Mdm2 inhibitor nutlin-3 caused the accumulation of p53 (Supplementary Figures S3 and S7a). In addition, double-knockdown experiments indicate that p53 ubiquitination is Mdm2-dependent in cells depleted of S5a (Supplementary Figure S7b).

To determine whether the differential effect on p53 and Mdm2 stability is simply a consequence of the degree of proteasome impairment that is achieved by knockdown of intrinsic subunits of the proteasome S9/PSMD11/Rpn6 and PSMB3/β3 were targeted. The former protein is a subunit of the 19S RP that is required for RP assembly.^61^ The latter protein is a component of the 20S core that is required for core assembly.^62^ Knockdown of S9 or PSMB3 stabilized both p53 and Mdm2 (Figure 2b). This shows that the differential effect on degradation of p53 and Mdm2 is selective for knockdown of S5a. It also confirms that the 19S RP and 20S core of the proteasome are required for degradation of both p53 and Mdm2.

These data indicate that there is a differential requirement for S5a in the proteasomal degradation of p53 and its E3 ligase Mdm2. The degradation of p53 but not Mdm2 being highly sensitive to depletion of S5a.

### The ubiquitin-interacting motifs of S5a are required for degradation of p53

**Figure 3:**
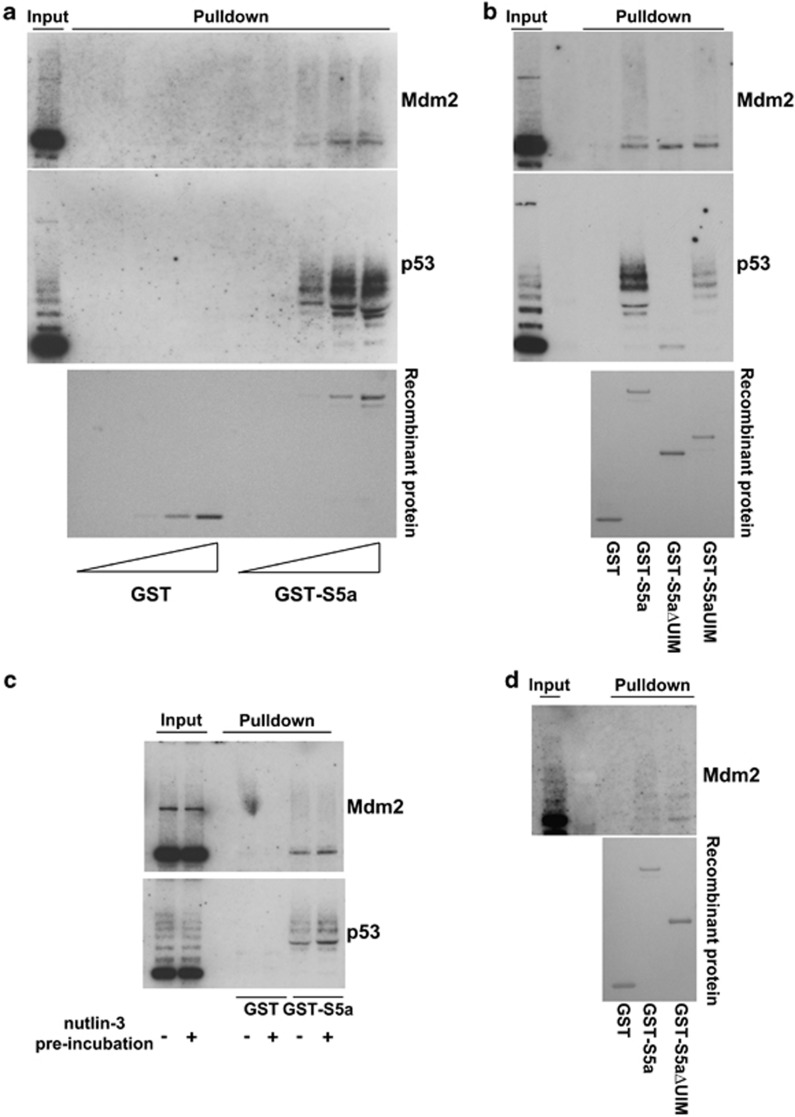
S5a can associate with p53 and Mdm2 through its UIMs. Pulldown experiments were carried out using lysates of bortezomib-treated cells as a source of correctly modified/ubiquitinated p53 and Mdm2. The inputs (10% of the lysate used per pulldown) and eluted material were analysed by western blotting using anti-Mdm2 antibody 4B2 and anti-p53 antibody DO.1. Recombinant proteins used in the pulldown experiments were analysed by SDS–polyacrylamide gel electrophoresis and Coomassie staining. (**a**) A375 lysates were incubated with 0.1, 0.3, 1, 3 and 10 μM GST-S5a or an equal amount of GST. High-molecular weight conjugates of p53 and Mdm2 were specifically pulled down by full-length S5a. (**b**–**d**) Lysates were incubated with 3 μM GST-S5a or an equal amount of the indicated GST-fusion. (**b**) GST-S5a and an N-terminal deletion (GST-S5aUIM), which retains both UIMs, associated with high-molecular weight conjugates of p53 and Mdm2. In contrast, a C-terminal deletion (GST-S5aΔUIM) that lacks both UIMs did not pulldown high-molecular weight conjugates of p53 or Mdm2. The association of S5a with conjugates of p53 and Mdm2 requires its UIMs. (**c**) Extracts were incubated with nutlin-3 (10 μM) for 1 h before GST-pulldown to interfere with the interaction between p53 and Mdm2. Nutlin-3 did not prevent the association of S5a with high-molecular weight conjugates of p53 or Mdm2. (**d**) GST-S5a associates with Mdm2 in HCT116 p53^−/−^ cells. The interaction between Mdm2 high-molecular weight conjugates and S5a does not require bridging by p53.

S5a contains an N-terminal von Willebrand factor A domain that is required for its association with the proteasome and two C-terminal ubiquitin-interacting motifs (UIMs). Purified S5a associated in a UIM-dependent manner with high-molecular weight conjugates of p53 (Figure 3). It was also observed that high-molecular weight conjugates of Mdm2 can complex with S5a independently of p53. This would appear to be at odds with the observation that Mdm2 degradation is insensitive to S5a knockdown. However, it is possible that there is loss of selectivity of S5a *in vitro* or that *in vivo* there are S5a-independent preferential or compensatory pathways for the proteasomal recruitment of Mdm2.

**Figure 4:**
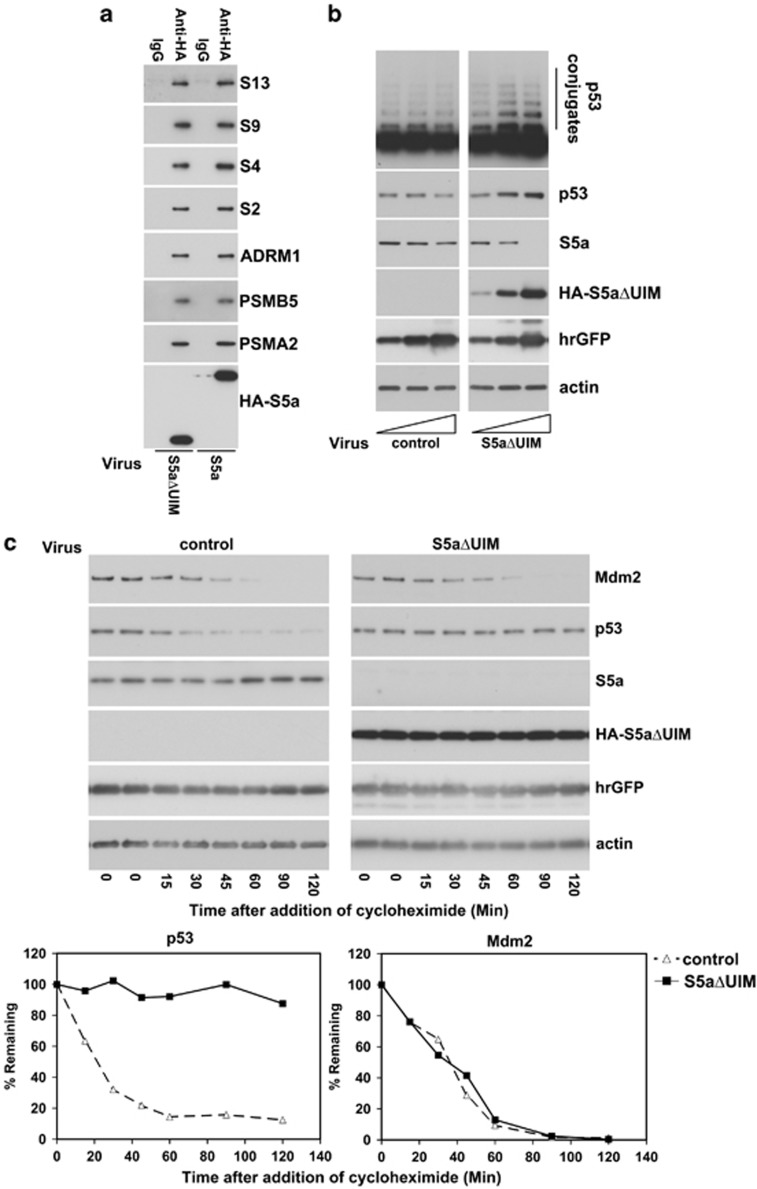
Overexpression of a mutant S5a lacking UIMs stabilizes p53 but not Mdm2. A375 cells were infected with an adenovirus expressing hrGFP alone (control) or a bicistronic adenovirus expressing hrGFP along with an HA-tagged deletion of S5a missing the UIMs (S5aΔUIM) or HA-tagged full-length S5a. (**a**) Cells were lysed under native conditions that maintain the integrity of the 26S proteasome. Lysates were immunoprecipitated with an irrelevant control immunoglobulin G or an anti-HA antibody. The immunoprecipitates were analysed by western blotting. The proteasomal subunits PSMA2/α2 and PSMB5/β5 (20S core), ADRM1/Rpn13, S2/PSMD2/Rpn1, S4/PSMC1/Rpt2 (19S base) and S9/PSMD11/Rpn6 and S13/PSMD14/Rpn11 (19S lid) were specifically co-immunoprecipitated with HA-tagged S5aΔUIM and S5a. This demonstrates that HA full-length S5a and S5aΔUIM are incorporated into the 26S proteasome. (**b**) A375 cell were infected with the indicated adenoviruses at MOI of 150, 500 and 1500. Fourty-eight hours after infection, protein expression was analysed by western blotting. An anti-HA antibody was use to detected S5aΔUIM as it does not contain the epitope recognized by the S5a antibody used. Expression of S5aΔUIM caused a decrease in the level of endogenous S5a. This could be due to a reduction in protein stability when endogenous S5a is prevented from associating with the proteasome. S5aΔUIM increased the level of p53 and p53 conjugates. (**c**) Adenovirus-mediated ectopic expression of S5aΔUIM interfered with the degradation of p53 but had no affect on the degradation of Mdm2.

A C-terminal deletion of S5a lacking its UIM which can still be incorporated into the proteasome has been used widely in lower organisms to assess the contribution of the ubiquitin-binding activity of S5a homologues to protein degradation.^5, 6, 16, 17, 63, 64^ A375 cells were infected with an adenovirus that produces an equivalent deletion of human S5a (S5aΔUIM) that lacks both UIMs. Co-immunoprecipitation of multiple subunits of the 19S RP and the 20S core with S5aΔUIM shows that the S5a deletion is incorporated into 26S proteasomes (Figure 4a). Expression of S5aΔUIM to a sufficient level to prevent the association of endogenous S5a with the proteasome would be predicted to act in a dominant-negative manner and block S5a UIM-dependent protein degradation. Overexpression of S5aΔUIM in A375 cells caused the accumulation of p53 and an increase in the level of high-molecular weight p53 conjugates (Figure 4b). Strikingly, overexpression of S5aΔUIM reduced the rate of p53 protein degradation without affecting the stability of Mdm2 (Figure 4c). This suggests that the UIMs of S5a are selectively required for the proteasomal degradation of p53. Ectopic expression of S5aΔUIM caused a dramatic decrease in the levels of endogenous S5a (Figures 4b and c). Failure to properly incorporate S5a into the 19S RP results in a decrease in S5a protein levels.^62^ It may be the case that endogenous S5a that is displaced from the 19S RP by S5aΔUIM is more unstable than the 19S RP bound protein.

**Figure 5:**
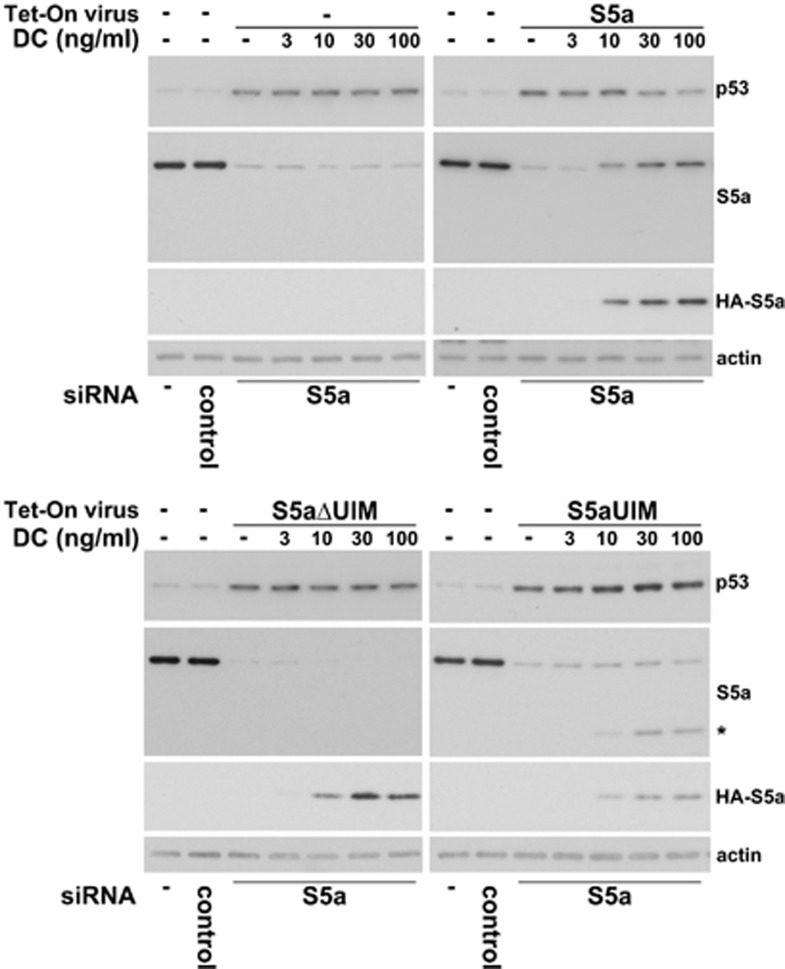
Wild-type S5a rescues the effect of S5a knockdown on p53 levels. A375 cells were mock infected (−) or infected with the indicated Tet-On adenovirus expressing siRNA-resistant, HA-tagged wild-type or mutant S5a. Where shown endogenous S5a was knocked down using siRNA S5a(A). Doxycycline (DC) was then added to the indicated concentrations to induce expression of the S5a constructs. Equivalent exposures of western blots are shown. The epitope recognized by the S5a antibody is deleted in S5aΔUIM. Consequently, an anti-HA antibody was use to compare the expression of the different S5a constructs. In mock-infected cells, DC had no effect on p53 or S5a protein levels. Despite being expressed to similar levels, wild-type S5a but not a mutant, where the UIMs of S5a are deleted (S5aΔUIM), partially rescued the increase in p53 expression resulting from knockdown of endogenous S5a. The UIMs of S5a are required for p53 degradation. The expression of S5a and (S5aΔUIM) caused a decrease in the remaining endogenous S5a. This indicates that a high proportion of the cells were infected with each adenovirus, and that endogenous S5a was displaced from the proteasome. A deletion of S5a lacking the proteasome-binding regions but containing the UIMs (S5aUIM) also failed to rescue p53 accumulation following endogenous S5a knockdown. The position of migration of S5aUIM is indicated by ‘*’. Instead, S5aUIM further increased p53 protein expression, presumably through sequestration of p53 away from the proteasome. Accumulation of p53 following S5a knockdown is not due to the ability of the UIMs of S5a to prevent forked chain ubiquitin formation. Expression of S5aUIM was lower than that for the other S5a constructs. This is consistent with destabilization due to exclusion from the proteasome.

To further investigate the role of the UIMs of S5a in the degradation of p53, the ability of ectopically expressed wild-type S5a and S5a mutants to rescue the effect of endogenous S5a knockdown was compared. A375 cells were infected with doxycycline-inducible adenoviruses that encode siRNA-resistant S5a constructs. The efficiency of infection was verified by immunofluorescence (Supplementary Figure S8). Partial restoration of wild-type S5a levels reduced p53 accumulation (Figure 5). This indicates that increased p53 expression is not due to an off-target effect of the S5a siRNA. In contrast, ectopic expression of S5aΔUIM to a comparable level did not rescue the effect of S5a knockdown on p53 accumulation. This confirms that the UIMs of S5a are required for the degradation of p53.

A deletion of S5a (S5aUIM) lacking the N terminus, which is required for incorporation into the proteasome^17, 39^ but retaining the UIMs, further increased p53 levels compared with S5a knockdown. This mutant is able to associate with high-molecular weight conjugates of p53 (Figure 3b). These observations are consistent with further stabilization of p53 through sequestration away from the proteasome by S5aUIM. In cell-free systems, S5a and S5aUIM can promote proteasomal degradation by preventing the formation of non-degradable forked ubiquitin chains.^19^ The inability of S5aUIM to rescue indicates that accumulation of p53 following S5a knockdown is not due to a role of the UIMs of S5a in preventing forked ubiquitin chain formation.

### Suppression of S5a activates p53

**Figure 6:**
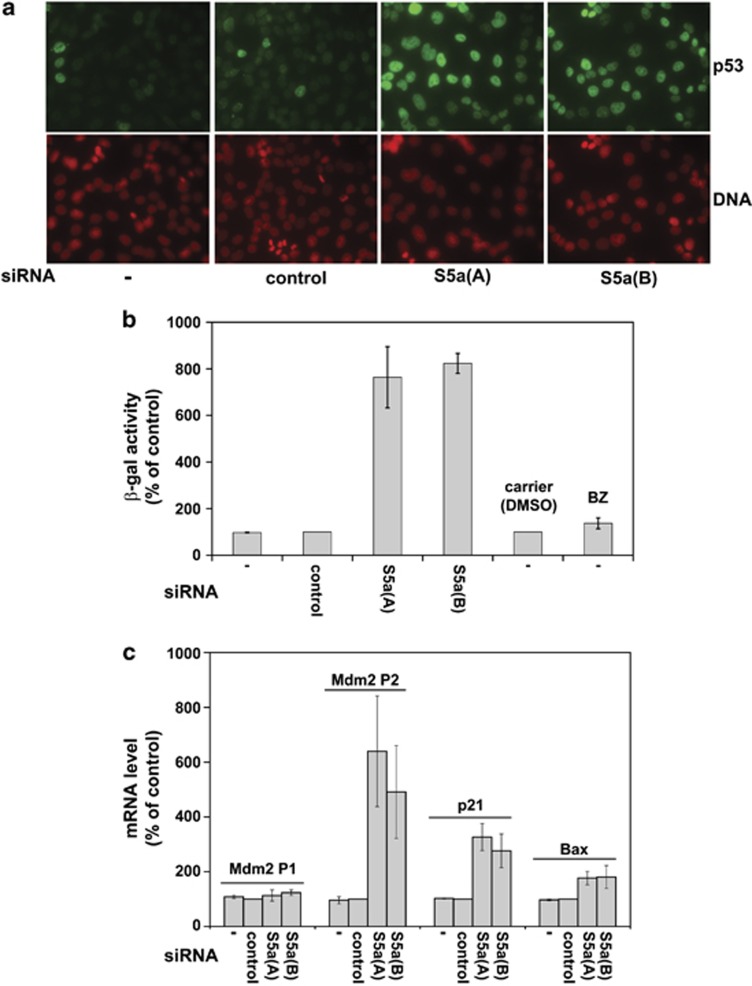
Knockdown of S5a causes the accumulation of p53 in the nucleus and increases p53-responsive transcript expression. A375 cells were mock transfected (−) or transfected with the indicated siRNA. (**a**) Cells were analysed by immunofluorescence using CM.1 to detect endogenous p53 (shown in green) and DNA was stained with DAPI (shown in red). Depletion of S5a caused an increase in the level of nuclear p53. (**b**) S5a knockdown increased the activity of a p53-responsive reporter driving the expression of β−galactosidase to a greater extent than proteasome inhibition by 100 nM bortezomib (BZ). β-galactosidase activity was normalized to total protein levels and expressed as a percentage of control (non-targeting siRNA). The values are mean±s.d. of three determinations. (**c**) siRNA-mediated suppression of S5a caused an increase in the expression of p53 target genes *p21* and *Bax,* and in the p53-responsive Mdm2 P2 transcript. Levels of the p53-independent P1 Mdm2 transcript were unaffected. mRNA expression was quantified by real-time PCR. mRNA levels were normalized to TATA box binding protein (TBP) and expressed as a percentage of control (non-targeting siRNA). The values are mean±s.d. of three experiments.

**Figure 7:**
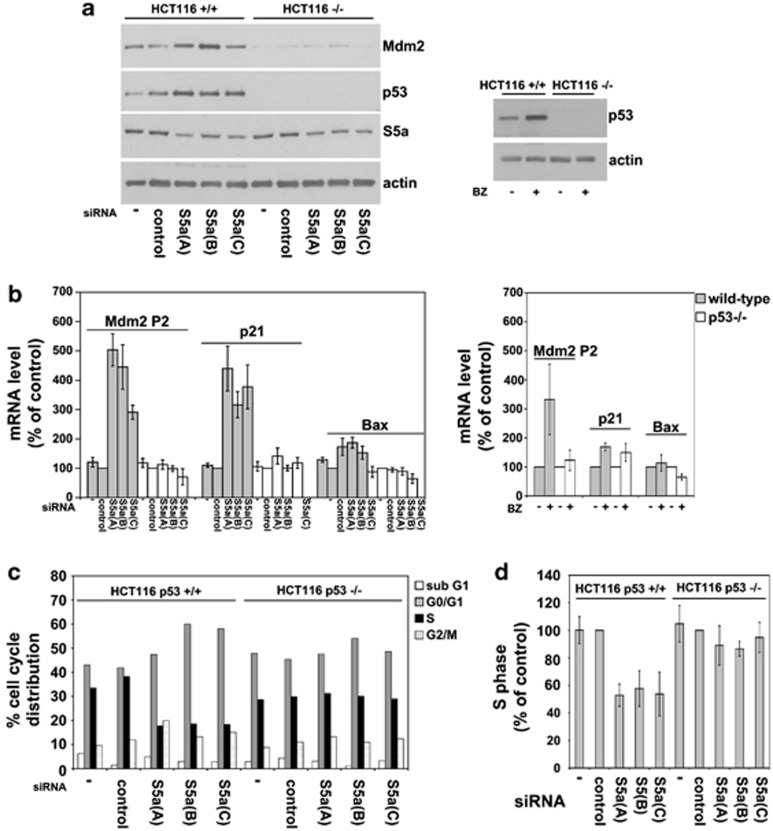
p53 activation due to S5a knockdown can inhibit proliferation. HCT116 p53^+/+^ and p53^−/−^ cells were transfected with siRNA or incubated with 50 nM bortezomib (BZ) as shown. (**a**) Protein expression was analysed by western blotting. The extent of S5a knockdown was similar in the two cell lines. Suppression of S5a caused an increase in p53 and Mdm2 protein levels in HCT116 p53^+/+^ cells. The increase in Mdm2 protein expression was p53-dependent. Proteasome inhibition by BZ caused a comparable increase in p53 protein levels. (**b**) Depletion of S5a caused a p53-dependent increase in the mRNA expression of the p53 target genes *Mdm2*, *p21* and *Bax*. BZ caused a p53-dependent increase in Mdm2 P2 mRNA levels but not p21 or Bax. S5a knockdown is more efficient than proteasome inhibition at increasing the transcriptional activity of p53 towards *p21* and *Bax*. mRNA levels were quantified by real-time PCR. mRNA levels were normalized to TATA box binding protein (TBP) and expressed as a percentage of control (non-targeting siRNA). The values are mean±s.d. of four experiments. (**c**, **d**) Seventy-two hours after transfection with the indicated siRNA, HCT116 cells were pulse labelled with BrdU and analysed by flow cytometry. Knockdown of S5a causes a p53-dependent decrease in the proportion of cells in S phase. (**c**) A representative experiment is shown. (**d**) The proportion of cells in S phase was also expressed as a percentage of control (control siRNA transfection in the particular cell line). The values are mean±s.d. of four experiments.

In A375 cells, knockdown of S5a results in the accumulation of p53 in the nucleus (Figure 6a) and causes an increase in p53-responsive transcriptional reporter activity (Figure 6b). The *Mdm2* gene is a transcriptional target of p53. Consistent with specific transcriptional activation of p53, knockdown of S5a increased the level of the p53-responsive Mdm2 P2 mRNA but had no affect on the level of p53-independent Mdm2 P1 mRNA (Figure 5c). In addition, S5a depletion increased mRNA expression of the p53 target genes *p21* and *Bax*. HCT116 p53^+/+^ and p53^−/−^ cells were used to determine the p53 dependency of the effects of S5a knockdown. S5a depletion resulted in a p53-dependent increase in Mdm2 P2, p21 and Bax mRNA expression (Figure 7b). Suppression of S5a increased Mdm2 protein levels in HCT116 p53^+/+^ cells. However, little or no increase in Mdm2 protein expression was observed in HCT116 p53^−/−^ cells following S5a knockdown (Figure 7a). Similar results were observed in matched control MCF7 cells, and MCF7 cells stably transfected with dominant-negative truncated p53 (DD-p53) that attenuates the activity of endogenous p53 (Supplementary Figure S9). The p53 target gene mRNA induction following S5a knockdown is similar to that observed with p53-activating agents such as doxorubicin and 5-fluorouracil.^37, 65^ These data show that S5a knockdown increases p53 transcriptional activity, and that the increase in Mdm2 protein expression following the suppression of S5a is due to the transcriptional activation of p53.

Bortezomib treatment caused p53 accumulation (Figure 1 and Figure 7a) but a relatively small increase in p53-responsive transcriptional reporter activity, (Figure 6b) and it did not result in a p53-dependent increase in p21 or Bax mRNA levels (Figure 7b). This confirms that inhibition of the proteolytic activity of the proteasome does not efficiently increase the transcriptional activity of p53. However, bortezomib did cause a p53-dependent increase in Mdm2 P2 mRNA, indicating, as observed previously,^66^ that proteasome inhibition can have p53 target gene-selective effects. The use of a combination of bortezomib and nutlin-3 indicates that the transcriptional activity of p53 can be suppressed by Mdm2 in cells in which the proteolytic activity of the proteasome is inhibited (Supplementary Figure S10).

The effect of S5a knockdown on the cell cycle was investigated in HCT116 cells. S5a knockdown caused a p53-dependent decrease in the proportion of cells in S phase (Figures 7c and 7d). This demonstrates that p53 can have an important role in reducing cell proliferation following the depletion of S5a.

## Discussion

Our study shows that the ubiquitin receptor S5a participates in the recognition of ubiquitinated p53 by the proteasome. Suppression of S5a increases p53 protein stability and causes the accumulation of ubiquitinated p53. Furthermore, the UIMs of S5a are required for p53 degradation. In contrast, degradation of Mdm2 by the proteasome is unaffected by interfering with S5a. This demonstrates that there is a difference in the pathways of proteasomal recognition of p53 and its major E3 ligase Mdm2. Through selective effects on the stability of p53 and Mdm2, targeting S5a can result in more efficient transcriptional activation of p53 than general proteasome inhibition.

Intrinsic proteasomal S5a may have the major role in p53 recognition. However, a significant proportion of S5a exists free of the proteasome.^16, 17, 18, 19^ It is thus possible that extraproteasomal S5a participates in bringing ubiquitinated p53 to the proteasome. Targeting of p53 to S5a could involve the interaction of ubiquitinated p53 with a shuttle receptor. S5a binds directly to polyubiquitin but can also bind to the UBL domain of UBA/UBL adaptor proteins through the same UIMs. There is evidence both for and against a role of the UBA/UBL adaptor proteins hHR23A and hHR23B in the degradation of p53.^40, 41, 42, 43^ Mdm2 can promote the ubiquitin-independent association of p53 with the proteasome.^46^ There may be handover of ubiquitinated p53 from Mdm2 to S5a. Intriguingly, ubiquitination of p53 causes a decrease in the affinity of the interaction between p53 and Mdm2.^67^ This could mediate the release of p53 from Mdm2 in order to transfer it to S5a.

S5a is not essential for Mdm2 degradation by the proteasome. Mdm2 maintains low levels of p53 in all the cell lines used,^58, 59, 60^ and we observed that it is required for the accumulation of ubiquitinated p53 when S5a is knocked down. This shows for the first time that S5a can be selectively involved in the recognition of an E3 ligase and its substrate by the proteasome. S5a depletion had a smaller effect on the general pattern of high-molecular weight ubiquitin conjugates than inhibition of the proteolytic activity of the proteasome, further indicating that targeting S5a in human cells has selective effects on protein degradation by the proteasome. These observations are consistent with the finding that in *S. cerevisiae*, Rpn10, the yeast homologue of S5a, is required for the proteasomal degradation of only a subset of ubiquitinated proteins.^5^ In pulldown experiments, high-molecular weight conjugates of Mdm2 associate with S5a. This may be due to loss of specificity *in vitro*. However, it is possible that recruitment pathway selectivity is determined upstream of S5a at the level of ubiquitin-binding shuttle proteins. The selective interaction of Mdm2 with a shuttle protein may result in its targeting to the proteasome by an S5a-independent mechanism. Alternatively, there may be redundancy in the pathways of proteasomal recognition of Mdm2, and an additional receptor could compensate for the loss of S5a.

Variations in the nature of the ubiquitin tag including the degree of polyubiquitination and the type of inter-ubiquitin crosslinks could provide the biochemical basis for differences in the pathways of recruitment of p53 and Mdm2. A contributory factor to differences in the ubiquitin tag could be that under at least some circumstances, endogenous Mdm2 ubiquitination is not mediated by autoubiquitination but rather by other E3 ligases.^68, 69, 70^ Multiple types of ubiquitin modification can target proteins for degradation by the proteasome.^1^ Ubiquitin-binding proteins can display selectivity for ubiquitin chains of different lengths and for ubiquitin chains generated through the linking of different lysine residues.^21, 71, 72^ There may also be a direct interaction between the substrates and the receptor or another appropriately positioned subunit associated with the proteasome that favours binding to a particular ubiquitin receptor. The conjugation of other UBL modifiers to p53 or Mdm2 could also influence the pathway of recognition.^73^ Several deubiquitinating enzymes associate with the proteasome^74^ and could have selective effects on substrate recognition.

Targeting S5a resulted in the transcriptional activation of p53 and can cause a p53-dependent decrease in cell proliferation. Stabilization of p53 without effects on the stability of Mdm2 provides a mechanism for this increase in the activity of p53. This alters the set point of the p53:Mdm2 feedback loop. The balance between levels of p53 and Mdm2 has an important influence on tumour suppression and the response of p53 to stress. By providing a means to alter the relative stability of p53 and Mdm2, differences in recognition of p53 and Mdm2 by the proteasome could be important in p53 regulation. Modulation of S5a or additional ubiquitin receptors selectively involved in the recognition of p53, and Mdm2 could participate in the control of p53 during tumour development and by cellular signals. S5a levels are increased in cancer.^75, 76, 77^ The expression and splicing of S5a is developmentally regulated.^17, 78^ The ubiquitin/UBL-binding activity of S5a homologues is controlled by stress-dependent mono-ubiquitination in lower organisms.^18, 79^ S5a is one of three 19S RP subunits that are degraded in a caspase-dependent manner in response to pro-apoptotic agents, possibly as part of a feed forward pro-apoptotic response.^80^ Ubiquitin receptors are known to be involved in DNA damage responses, members of the UBA/UBL family are DNA damage inducible.^81^

Bortezomib, an inhibitor of the proteolytic activity of the proteasome is used in the treatment of multiple myeloma and mantle cell lymphoma. Work is underway to extend the use of bortezomib to other cancers including solid tumours. There are problems with *de novo* and acquired resistance, and also with dose-limiting toxicities of bortezomib.^82^ Interfering with protein degradation by targeting S5a may be of therapeutic benefit. In some cell types, p53 can contribute to the anti-tumour activity of bortezomib.^83^ However, inhibition of the proteolytic activity of the proteasome can stabilize p53 without efficiently increasing its transcriptional activity. A reason for this is the stabilization of Mdm2 that causes the accumulation of sufficient Mdm2 to repress p53 by direct binding.^30, 35, 37^ Owing to the selective stabilization of p53 and not Mdm2 targeting S5a would have the advantage over inhibitors of the proteolytic activity of the proteasome of more robust transcriptional activation of p53. Interference with the degradation of only a subset of proteasomal substrates may result in a wider therapeutic window for targeting S5a. In addition, targeting the proteasome in alternative ways, such as antagonizing the ubiquitin/UBL-binding activity of S5a, could overcome resistance to bortezomib. It would be of interest to identify selective inhibitors of ubiquitin receptors. In support of this possibility, there are significant variations in the amino-acid residues in ubiquitin required for interacting with different receptors.^71, 84, 85^ In addition, it may be possible to identify antagonists that bind receptors at regions away from the direct site of ubiquitin interaction.

This study shows that the ubiquitin receptor S5a can have a selective role in the proteasomal degradation of ubiquitinated proteins in human cells. S5a is required for the degradation of p53 but not its E3 ligase Mdm2. Additional work is required to completely define the pathways of proteasomal recognition of p53 and Mdm2, and to determine the contribution of ubiquitin receptor regulation to the control of p53. It would also be of interest to further investigate the therapeutic potential of targeting S5a and any additional ubiquitin receptors that co-operate with S5a in the recognition of p53 by the proteasome.

## Materials and methods

### Cell culture

A375, MCF7 and HCT116 cells were cultured as described previously.^37, 49^ Cells were seeded onto six-well plates: A375 (6 × 10^4^ cells/well), MCF7 (2 × 10^5^ cells/well) and HCT116 (0.5–1 × 10^5^ cells/well). Inhibitors: bortezomib, (LC Laboratories, Woburn, MA, USA); nutlin-3, (Cayman Chemicals, Ann Arbor, MI, USA).

### Western blotting

Unless otherwise indicated, cell extracts were prepared by direct lysis into SDS–urea electrophoresis sample buffer and western blotting was carried out as described previously.^49^ Peroxidase-conjugated secondary antibodies were supplied by Jackson ImmunoResearch Laboratories (Suffolk, UK) and used at a dilution of 1/10 000.

Antibodies: Mdm2 (4B2) and p53 (DO.1 and CM.1), Moravian-Biotechnology (Brno, Czech Republic); p53 (SAPU), Scottish Antibody Production Unit (Carluke, Scotland); actin (Ab-1, JLA20) and ubiquitin (P4D1, 05-944), Millipore (Watford, UK); S5a/PSMD4/Rpn10 (S5a-18, PW9250) and PSMB5/β5 (PW8895), Enzo Life Sciences (Exeter, UK); S5a (14899-1-AP), ADRM1/Rpn13 (11468-1-AP), S2/PSMD2/Rpn1 (14748-1-AP), S4/PSMC1/Rpt2 (11196-1-AP), S9/PSMD11/Rpn6 (14786-1-AP) and S13/PSMD14/Rpn11 (12059-1-AP), Proteintech Group (Manchester, UK); ADRM1/Rpn13 (ab56852), HA (HA.C5, ab18181) and H2AX (ab11175), Abcam (Cambridge, UK); GFP (7.1 and 13.1, 11814460001), Roche (Burgess Hill, UK); PSMA2/α2 (H-120, sc-67339), Santa Cruz (Heidelberg, Germany); phospho Ser-15 p53 (9284) and phospho Ser-139 histone H2AX (2577), Cell Signaling Technology (Hitchin, UK).

### Synthetic siRNA duplexes and transfections

ON-TARGETplus modified siRNA were purchased from Thermo Fisher Scientific (Epsom, UK). S5a(A): 011365-05, S5a(B): 011365-07, S5a(C): 011365-06, S9: 011367-10, PSMB3: 017489-07, Mdm2(A): 003279-12 and Mdm2(B): 003279-13. Transfection with synthetic siRNA duplexes (30 nM) was carried out using Oligofectamine or Lipofectamine RNAiMAX (Life Technologies, Paisley, UK) according to the manufacturer’s instructions. Cells were harvested 48 h post transfection, unless otherwise indicated.

### Pulldown experiments

Cells were treated with bortezomib (50 nM) for 6 h, washed twice in PBS and lysed in buffer 1: 20 mM HEPES (pH 7.5), 0.1% NP-40, 100 mM NaCl, 5 mM EDTA, 10 mM NaF, 10 mM Na-pyrophosphate, 10 mM β-glycerophosphate, complete protease inhibitor cocktail (Roche, Welwyn Garden City, UK) containing 10 mM NEM. This concentration of NEM was found to be the minimum required to maintain p53 ubiquitination over the course of the assay. Lysis was carried out on ice for 20 min with occasional vortexing and the lysates were centrifuged (14 000 *g*, 15 min, 4 °C). The unreacted NEM in the supernatants was neutralized by the addition of DTT to a final concentration of 10 mM. The supernatants (350 μl total volume) were incubated for 2 h at 4 °C with the indicated concentration of bacterially expressed GST or GST-fusion protein pre-bound to 20 μl of glutathione-sepharose beads (GE Healthcare, Little Chalfont, UK). The beads were washed three times in buffer 1 containing 1 mM DTT and eluted in SDS–urea sample buffer.

### Adenovirus infection

Adenoviruses were generated expressing C-terminally HA-tagged: wild-type S5a, S5aΔUIM (residues 1 to 195 of S5a, which lacks both UIMs) and S5aUIM (residues 196–377 of S5a that lacks the proteasome-binding region but contains both UIMs). Adenoviruses were constructed using the AdEasy system (Agilent Technologies, Stockport, UK) or Tet-On 3G Inducible Adeno-X Adenoviral System 3 (Clontech, Saint-Germain-en-Laye, France). Where necessary siRNA-resistant forms of S5a were generated by introducing silent mutations at positions 8 (A to G) and 11 (G to C) of the siRNA S5a(A) target sequence.

For overexpression experiments, the normal cell growth medium was replaced with medium containing 2% foetal bovine serum, and cells were infected with AdEasy viruses at a multiplicity of infection (MOI) of 1000 unless otherwise indicated. After 4 h, cells were washed twice in PBS and returned to normal growth medium. Cells were harvested 40 h after infection. For siRNA rescue, experiments cells were infected with adenoviruses at a MOI of 2000, 6–8 h after plating. After 16 h, siRNA transfection was carried out using Lipofectamine RNAiMAX. Four hours later, doxycycline was added to the indicated concentrations. Cells were harvested 40 h after doxycycline addition.

### Immunoprecipitation

For proteasome immunoprecipitation, cells were washed twice in PBS and lysed in buffer 2: 20 mM Tris (pH 7.5), 0.1% NP-40, 5 mM MgCl_2_, 10 mM NaF, 10 mM Na-pyrophosphate, 10 mM β-glycerophosphate, 20% glycerol, 2 mM ATP, 1 mM DTT, complete protease inhibitor cocktail Roche. ATP and glycerol contribute to maintaining the integrity of the 26S proteasome. Lysis was carried out on ice for 15 min with occasional vortexing. The lysates were centrifuged (14 000 *g*, 15 min, 4 °C). The pooled supernatants from one six-well plate (total volume 350 μl) were then incubated for 1 h at 4 °C with agarose beads covalently coupled to control immunoglobulin G, anti- PSMA2 antibody (MCP21, PW8335, Enzo Life Sciences) or anti-HA antibody (26181, Thermo Fisher Scientific). The beads were washed three times with buffer 2 and eluted in SDS–urea sample buffer. To detect ubiquitination of endogenous p53 cells were lysed under denaturing conditions and immunoprecipitation of p53 carried out as described previously.^49^

### Immunofluorescence

To detect p53, cells were fixed with ice-cold methanol–acetone and staining was carried out as described previously.^37^

### Quantitative detection of β-galactosidase

The assay was performed as described previously^49^

### RNA preparation and PCR

Total RNA was extracted using RNeasy columns (Qiagen, Crawley, UK), reverse transcription was carried out using random primers and quantitative PCR was performed as described previously.^49, 86, 87^

### Flow cytometry

Flow cytometry analysis of cells was performed as described previously.^88^
