# Supplementary material for: The degradation of p53 and its major E3 ligase Mdm2 is differentially dependent on the proteasomal ubiquitin receptor S5a
Source: Oncogene. 2013 Oct 14;33(38):4685–96. doi: 10.1038/onc.2013.413 (PMC4051618; doi:10.1038/onc.2013.413)
Supplement: Supplementary Information [file onc2013413x1.pdf]

The degradation of p53 and its major E3 ligase Mdm2 is differentially dependent on the proteasomal ubiquitin receptor S5a.

Alison Sparks, Saurabh Dayal, Joyutpal Das, Pamela Robertson, Sergio Menendez and Mark K. Saville.

Supplementary Information

Figure Legends

S1. S5a knockdown does not reduce the expression of other intrinsic proteasomal subunits. A375 cells were mock transfected (-) or transfected with the indicated siRNA. In a number of instances the knockdown of a proteasomal subunit can reduce the expression of additional subunits due to inhibition of subcomplex assembly. However, transfection with siRNA complementary to S5a selectively decreased S5a levels without reducing the protein expression of PSMA2/α2 and PSMB5/β5 (20S core), ADRM1/Rpn13, S2/PSMD2/Rpn1 and S4/PSMC1/Rpt2 (19S base) and S9/PSMD11/Rpn6 and S13/PSMD14/Rpn11 (19S lid).

S2. 26S proteasomes can be assembled that are depleted of S5a. Knockdown of most intrinsic subunits cannot be used to investigate their specific function because this inhibits proteasome assembly. However, evidence from lower organisms indicates that this is not the case for homologues of S5a. Loss of S5a homologues can increase the ease of dissociation of subcomplexes of the proteasome at super-physiological salt concentrations during purification but during earlier stages of purification and in cells the

26S proteasome remains intact. There is also evidence that S5a homologues can be relatively loosely associated with the proteasome and that a population of 26S proteasomes exist that do not contain this subunit. 48 hours after transfection with the indicated siRNA A375 cells were lysed under native conditions which maintain the integrity of the 26S proteasome. Lysates were immunoprecipitated with an irrelevant control IgG or an antibody that recognizes the endogenous intrinsic 20S subunit PSMA2. (a) Lysates and (b) immunoprecipitates were analysed by western blotting. S5a knockdown did not reduce the levels of other proteasomal subunits. The extent of siRNA-mediated S5a depletion was similar in lysates and in immunoprecipitated proteasomes. S5a knockdown had no effect on the specific co-immunoprecipitation of PSMB5 (20S core), S4 (19S base) and S9 and S13 (19S lid) with PSMA2. This indicates that knockdown of S5a does not grossly interfere with 26S proteasome assembly.

S3. Depletion of S5a does not increase DNA damage signalling. Phosphorylation of histone H2AX resulting in the formation of γ-H2AX is commonly used to assess DNA damage. Multiple DNA damage-activated kinases phosphorylate p53 at Ser15. Phosphorylation at this site can consequently be used as an indicator of the activity of these kinases. The effects of S5a knockdown, the DNA damaging agent doxorubicin and the non-genotoxic p53 activator nutlin-3 were compared. DMSO carrier, nutlin-3 (10 μM for 10 hours) or doxorubicin (0.3 μM for 6 hours) were added to A375 cells. As anticipated doxorubicin induced the phosphorylation of H2AX and of p53 at Ser15. In contrast depletion of S5a and treatment with the non-genotoxic p53 activator nutlin-3 did

not increase the level of γ-H2AX or increase the ratio of Ser15 phosphorylated p53 to total p53. This indicates that S5a knockdown does not activate DNA-damage pathways.

S4. S5a knockdown does not reduce the pool of free ubiquitin in ARN8 cells. A375 cells were mock transfected (-), transfected with non-targeting siRNA (control) or siRNA targeting S5a. Where indicated the proteasome inhibitor bortezomib (BZ: 50 nM) was added 7 hours prior to harvesting. Consistent with previous reports bortezomib caused a decrease in the level of unconjugated mono-ubiquitin and in the level of a 23 kDa ubiquitin conjugated protein. This is likely to be mono-ubiquitinated histone H2A, which is a major ubiquitinated protein in cells. We have observed that ubiquitinated histone H2A co-migrates with this band (data not shown). In contrast, S5a knockdown did not reduce the level of free mono-ubiquitin and it caused a less dramatic decrease in the 23 kDa ubiquitinated species.

S5. S5a knockdown selectively inhibits the degradation of p53 in additional cell lines. Cells were transfected with siRNA as shown and incubated with cycloheximide (20μg/ml) for the indicated time. Protein levels were analysed by western blotting (upper panels) and the western blots were quantified (lower panels). Different exposures of the westerns are shown so that protein levels in the absence of cycloheximide are matched. S5a knockdown stabilises p53 without affecting the degradation of Mdm2 in (a) MCF7 cells and (b) HCT116 cells.

S6. Mdm2 is degraded by the proteasome in cells depleted of S5a. MCF7 cells were transfected with the indicated siRNA, treated with carrier (-) or the proteasome inhibitor bortezomib (BZ, 50nM) as indicated for 4 hours before the addition of cycloheximide (CHX, 20μg/ml) in order to measure protein stability. Cells were harvested at the indicated times after the addition of cycloheximide. The upper panels show western blots. The lower panels show quantification of the western blots for p53 and Mdm2. For each individual condition the levels are expressed as a percentage of those at zero minutes. Values are the mean +/- range of two experiments. S5a knockdown and treatment with bortezomib stabilised p53 to a similar extent. S5a depletion had little or no affect on Mdm2 degradation. Bortezomib stabilised Mdm2 in cells expressing normal levels of S5a and in cells depleted of S5a. This indicates that Mdm2 is degraded by the proteasome under both circumstances.

S7. p53 is regulated by Mdm2 in A375 cells. (a) A375 cells were incubated for the indicated times with nutlin-3 (10μM) which interferes with the interaction between Mdm2 and p53. Inhibition of Mdm2 results in a rapid increase in p53 levels. (b) A375 cells were transfected with the indicated combinations of siRNA. siRNA Mdm2(A) and (B) are complementary to two different sequences in Mdm2. Where appropriate the total amount of siRNA was kept constant by the addition of control non-targeting siRNA. Knockdown of Mdm2 markedly reduces the level of high molecular weight p53 conjugates that accumulate following S5a knockdown. These data indicate that Mdm2 mediates the ubiquitination of p53 in cells depleted of S5a.

S8. S5a constructs are expressed in a high proportion of cells. A375 were transfected with siRNA S5a(A) and infected with Tet-On adenoviruses under identical condition to those used for siRNA rescue experiments (Figure 5). Doxycycline (DC:100ng/ml) was added as shown. Cells were fixed in 4% paraformaldehyde and analysed by immunofluorescence using HA.C5 to detect virally expressed HA-tagged wild-type and mutant S5a. All images are combined phase contrast and HA-immunofluorescence (shown in green).

S9. Knockdown of S5a activates p53 in MCF7 cells. MCF7 cells stably expressing dominant negative DDp53 (MCF7 DD) or matched control MCF7 cells stably expressing empty vector (MCF7 CMV) were mock transfected (-) or transfected with the indicated siRNA. DDp53 is a mini-protein consisting of the first 14 amino acids of p53 fused to the oligomerisation domain of p53. DDp53 interacts with the oligomerisation domain of endogenous p53, prevents its oligomerisation and consequently blocks its transcriptional activity. (a) The extent of S5a knockdown was similar in the two cell lines. Depletion of S5a resulted in an increase in p53 and Mdm2 protein expression in MCF7 CMV cells. The increase in Mdm2 protein level was p53-dependent. (b) S5a knockdown caused a p53-dependent increase in the mRNA expression of the p53 target genes *Mdm2* and *p21*. mRNA expression was quantified by real-time PCR. mRNA levels were normalised to TBP and expressed as a percentage of control (non-targeting siRNA). The values are means +/- S.D. of three experiments.

S10. The transcriptional activity of p53 can be suppressed by Mdm2 in cells treated with

bortezomib. A375 cells were incubated with the indicated concentration of bortezomib (BZ). After three hours nutlin-3 was added where shown. Cells were harvested six hours later. (a) Western blots (b) p53-reponsive transcriptional reporter assay. Nutlin increased reporter activity in the presence of bortezomib. In cells in which the proteolytic activity of the proteasome is inhibited, disruption of the interaction between p53 and Mdm2 can increase the transcriptional activity of p53.

S1

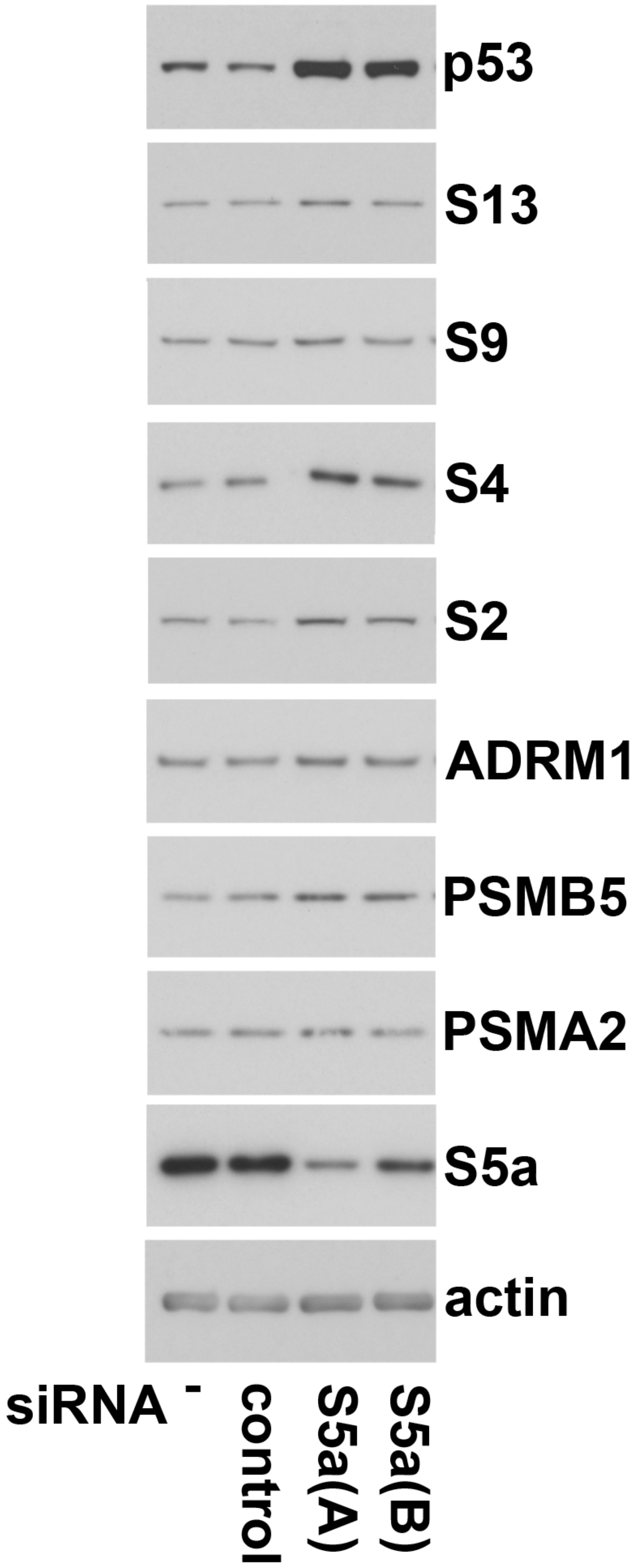
p53
S13
S9
S4
S2
ADRM1
PSMB5
PSMA2
S5a
actin
siRNA -
control
S5a(A)
S5a(B)

S2

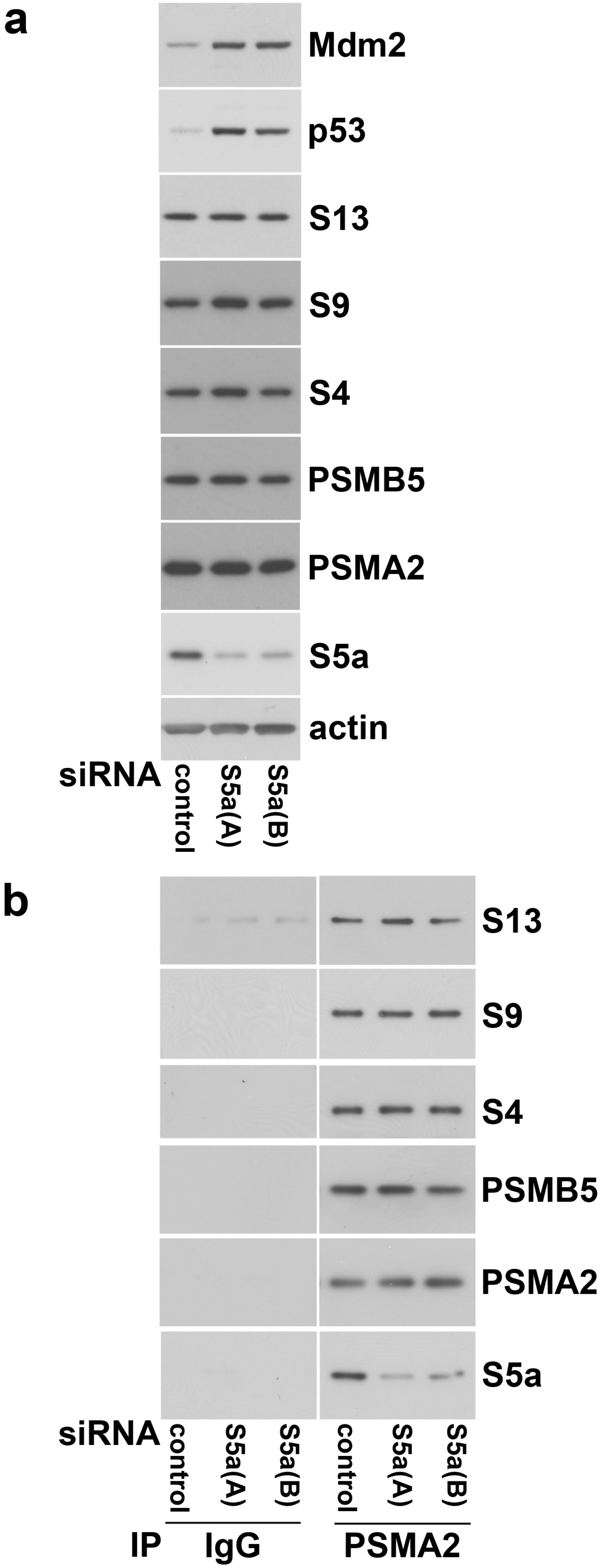
a
Mdm2
p53
S13
S9
S4
PSMB5
PSMA2
S5a
actin
siRNA
control
S5a(A)
S5a(B)
b
S13
S9
S4
PSMB5
PSMA2
S5a
siRNA
control
S5a(A)
S5a(B)
control
S5a(A)
S5a(B)
IP
IgG
PSMA2

S3

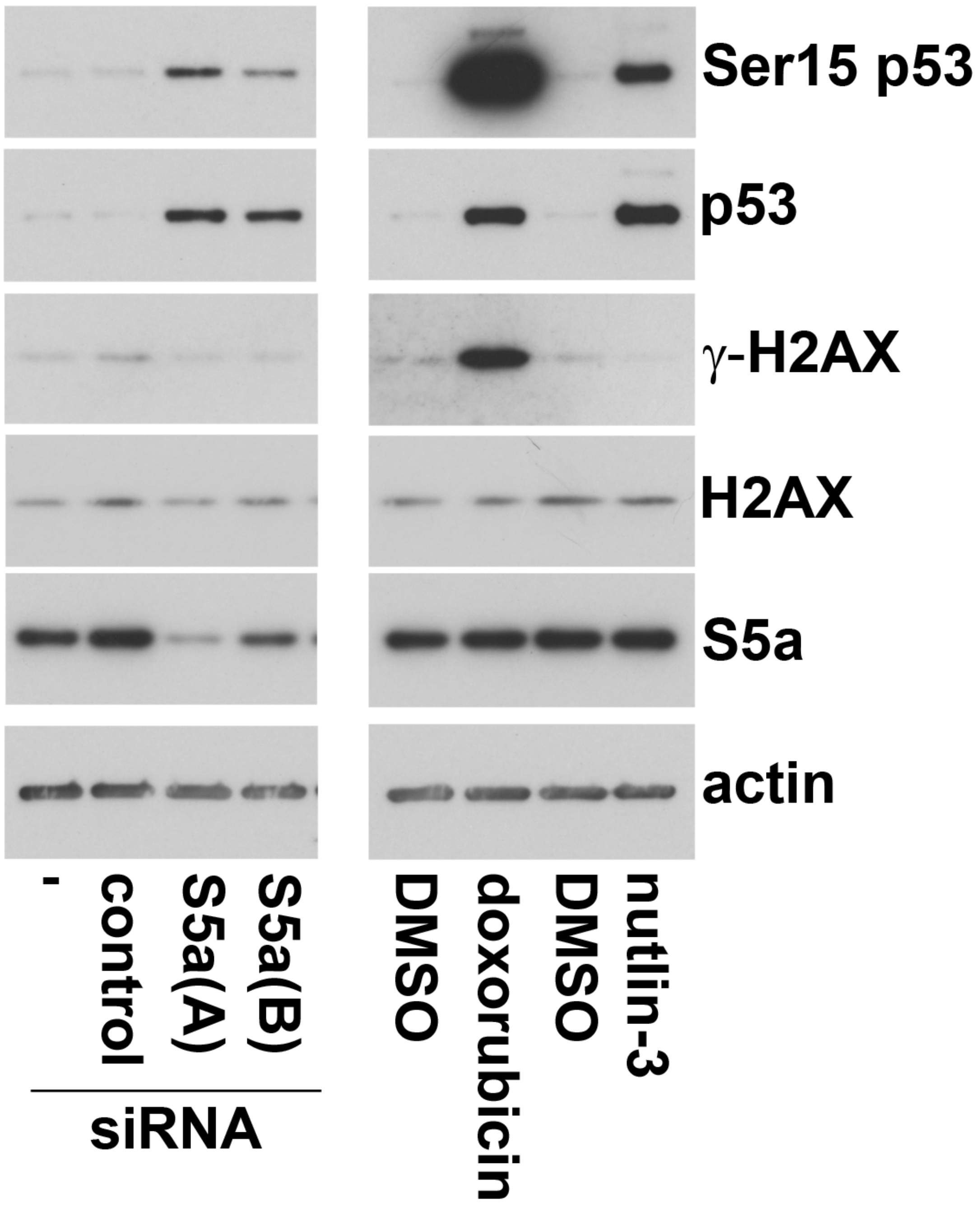
Ser15 p53
p53
γ-H2AX
H2AX
S5a
actin
-
control
S5a(A)
S5a(B)
siRNA
DMSO
doxorubicin
DMSO
nutlin-3

S4

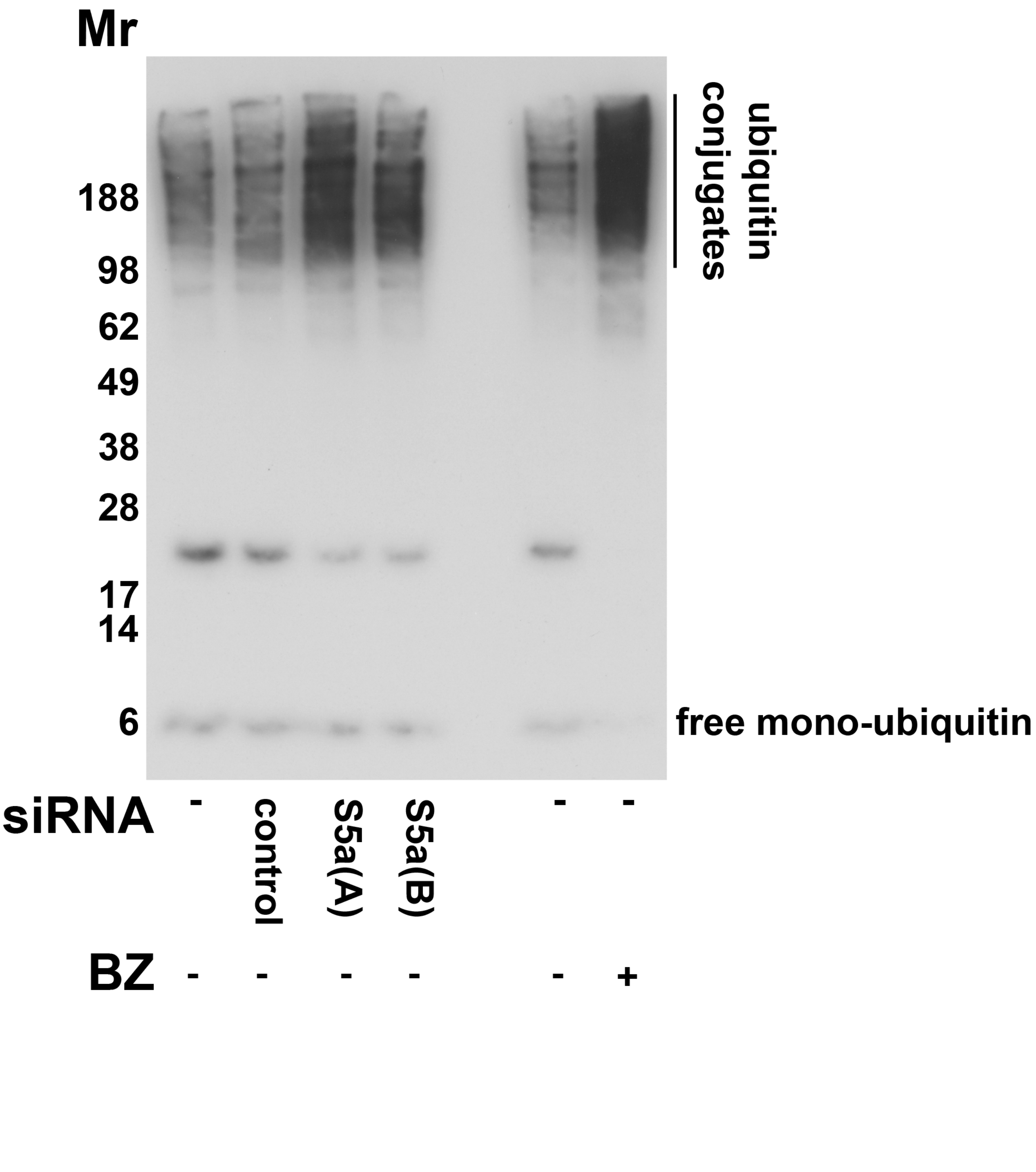
Mr
188
98
62
49
38
28
17
14
6
ubiquitin conjugates
free mono-ubiquitin
siRNA
-
control
S5a(A)
S5a(B)
-
-
BZ
-
-
-
-
-
+

S5

a MCF7 cells

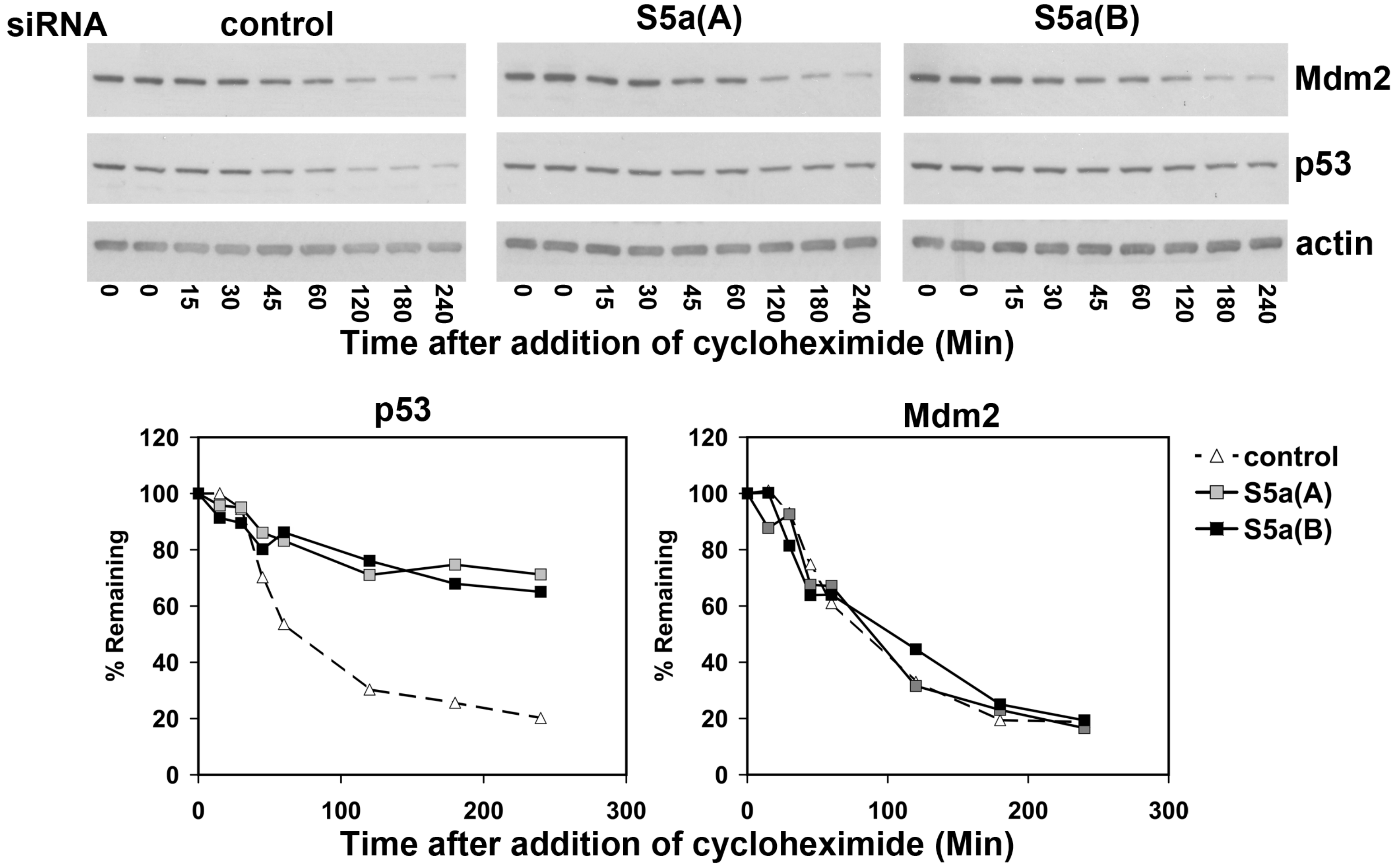

b HCT116 cells

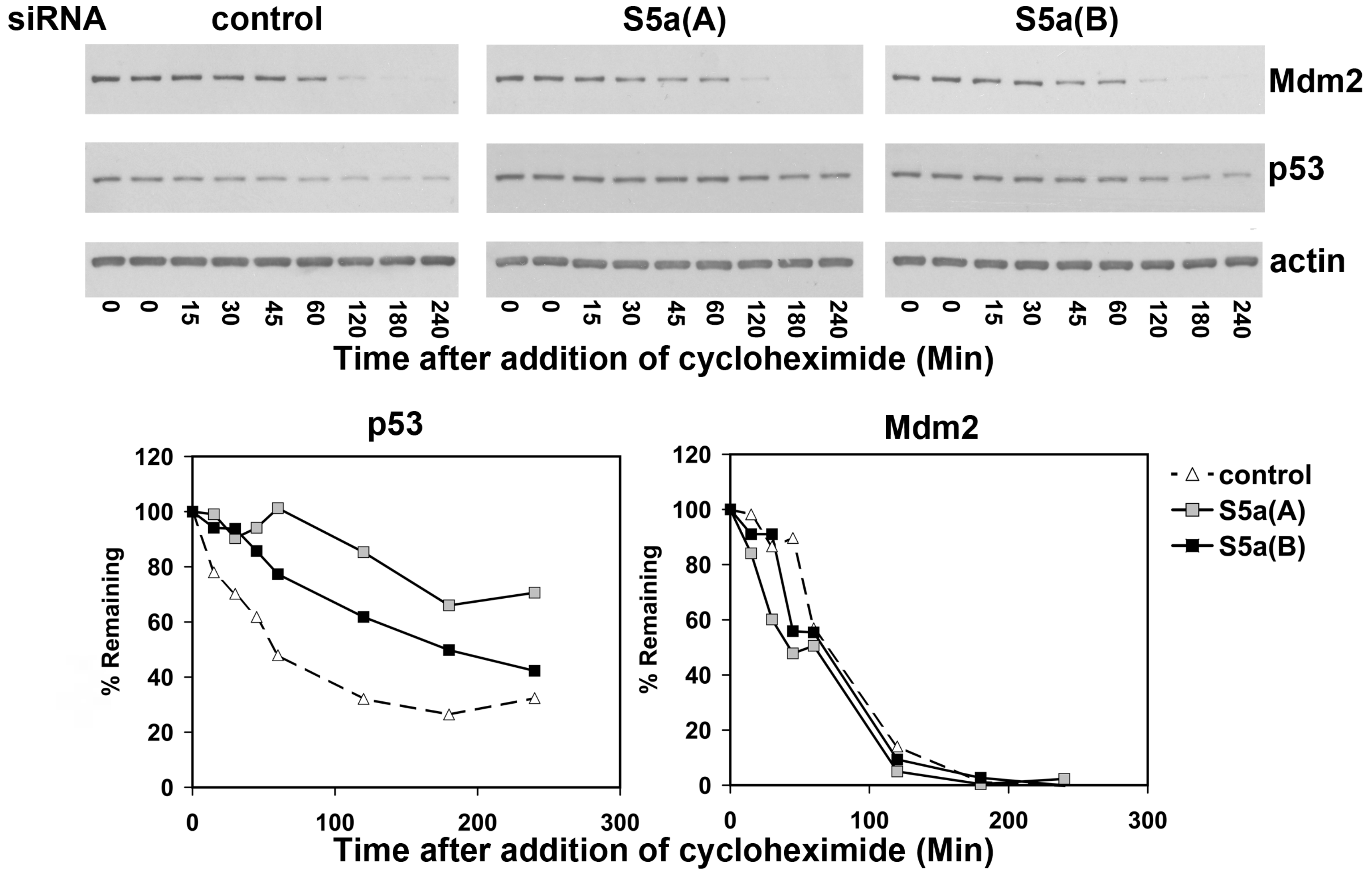

S6

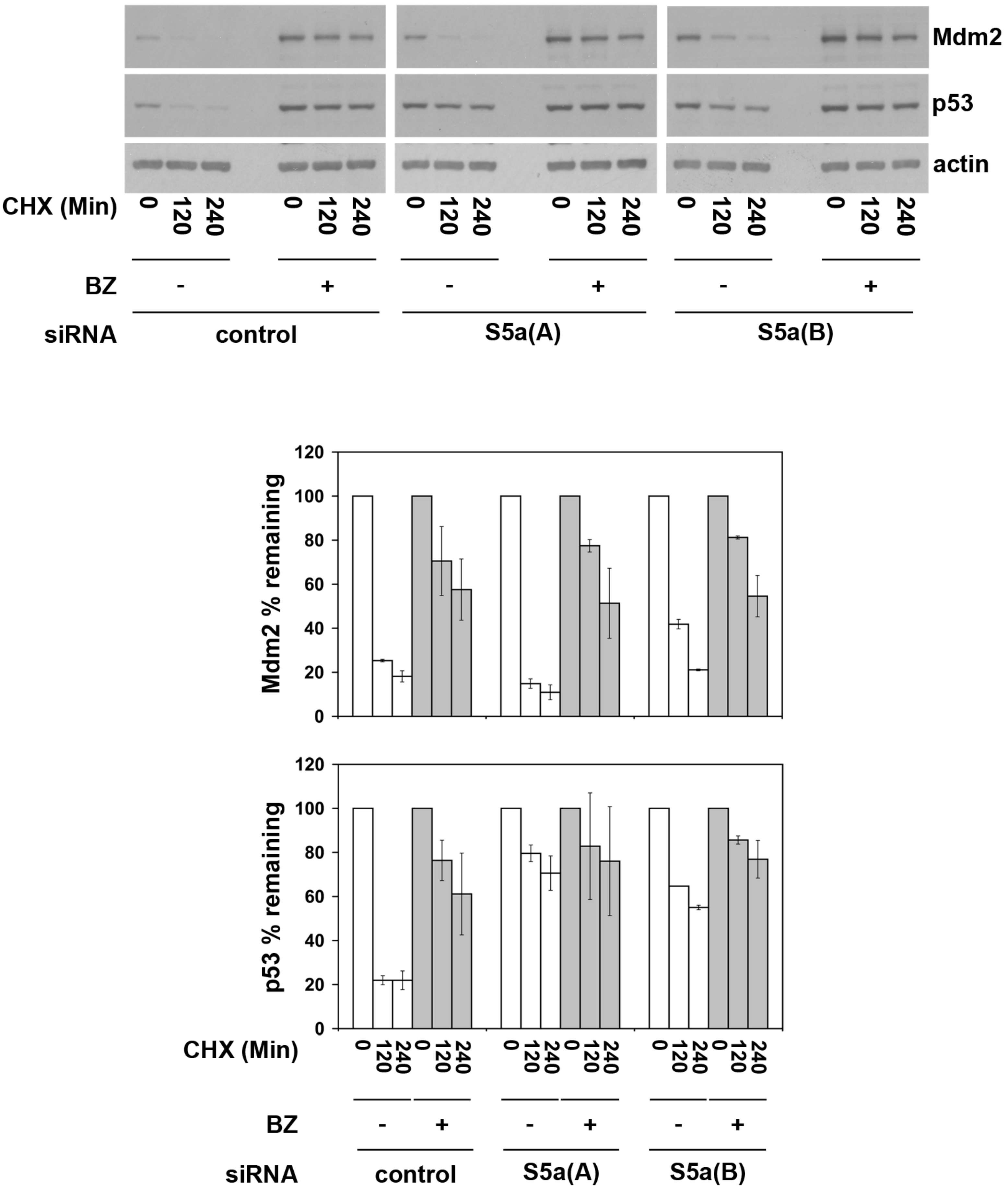
Mdm2
p53
actin
CHX (Min)
0 120 240 0 120 240 0 120 240 0 120 240 0 120 240 0 120 240
BZ
- + - + - +
siRNA
control
S5a(A)
S5a(B)
Mdm2 % remaining
p53 % remaining
0
20
40
60
80
100
120
CHX (Min)
0 120 240 0 120 240 0 120 240 0 120 240 0 120 240 0 120 240
BZ
- + - + - +
siRNA
control
S5a(A)
S5a(B)

S7

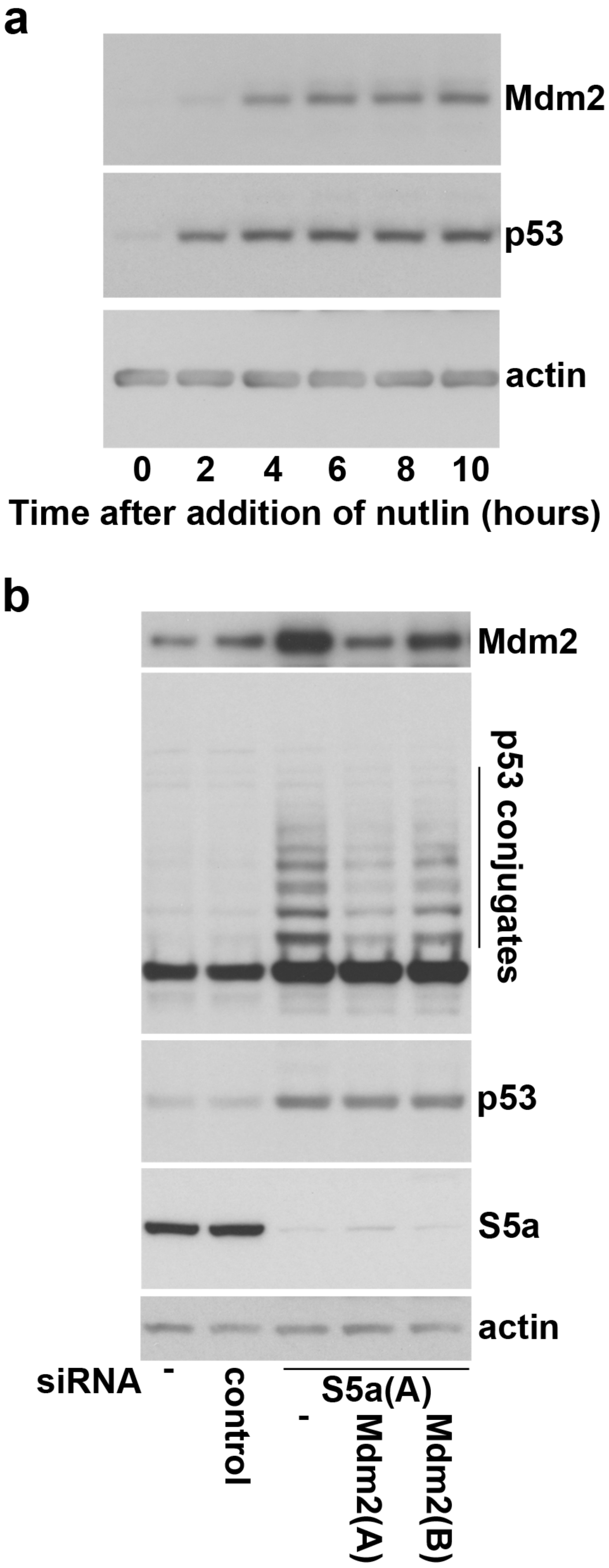
a
Mdm2
p53
actin
0
2
4
6
8
10
Time after addition of nutlin (hours)
b
Mdm2
p53 conjugates
p53
S5a
actin
siRNA
-
control
S5a(A)
-
Mdm2(A)
Mdm2(B)

S8

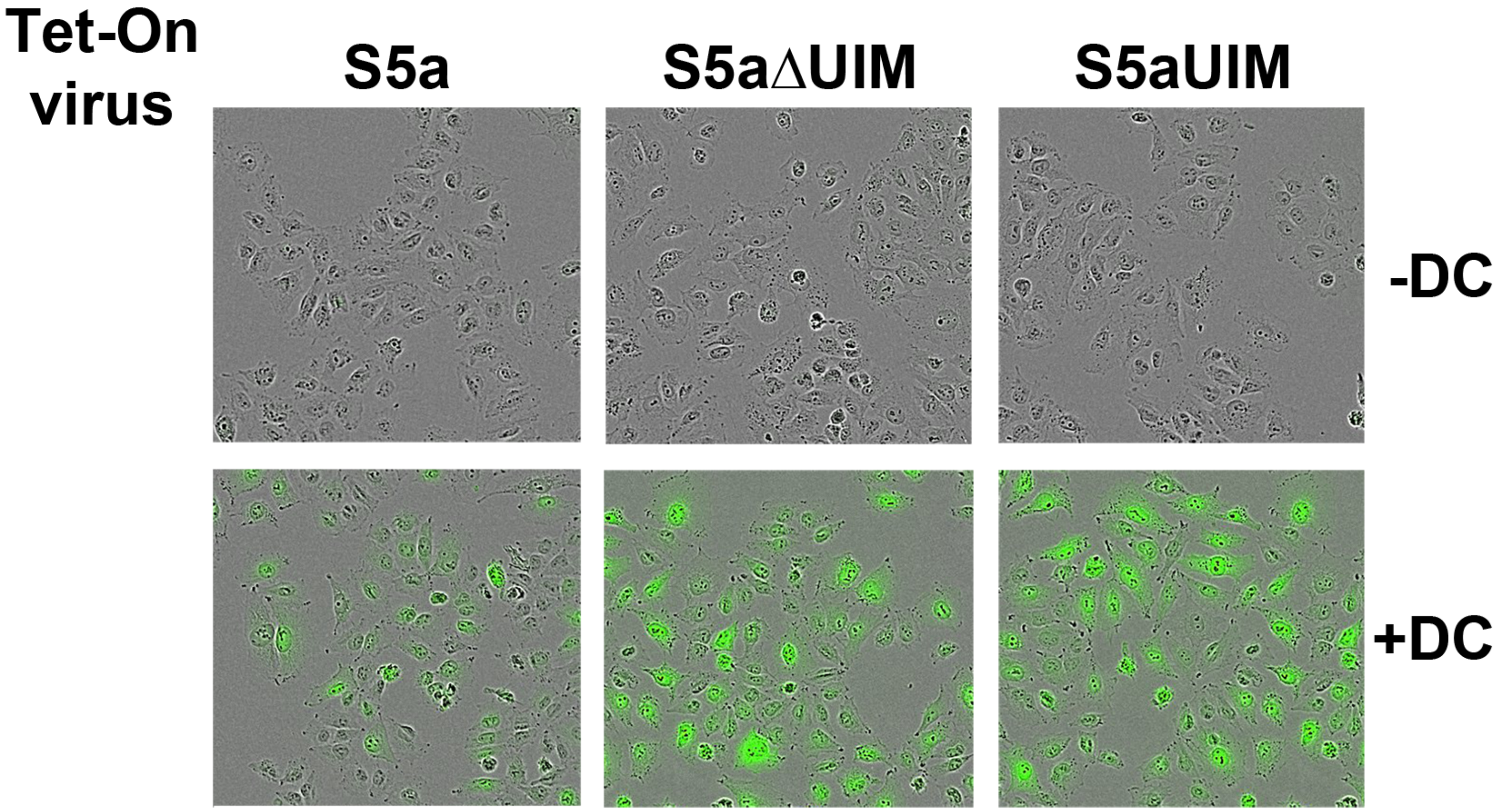

S9

a

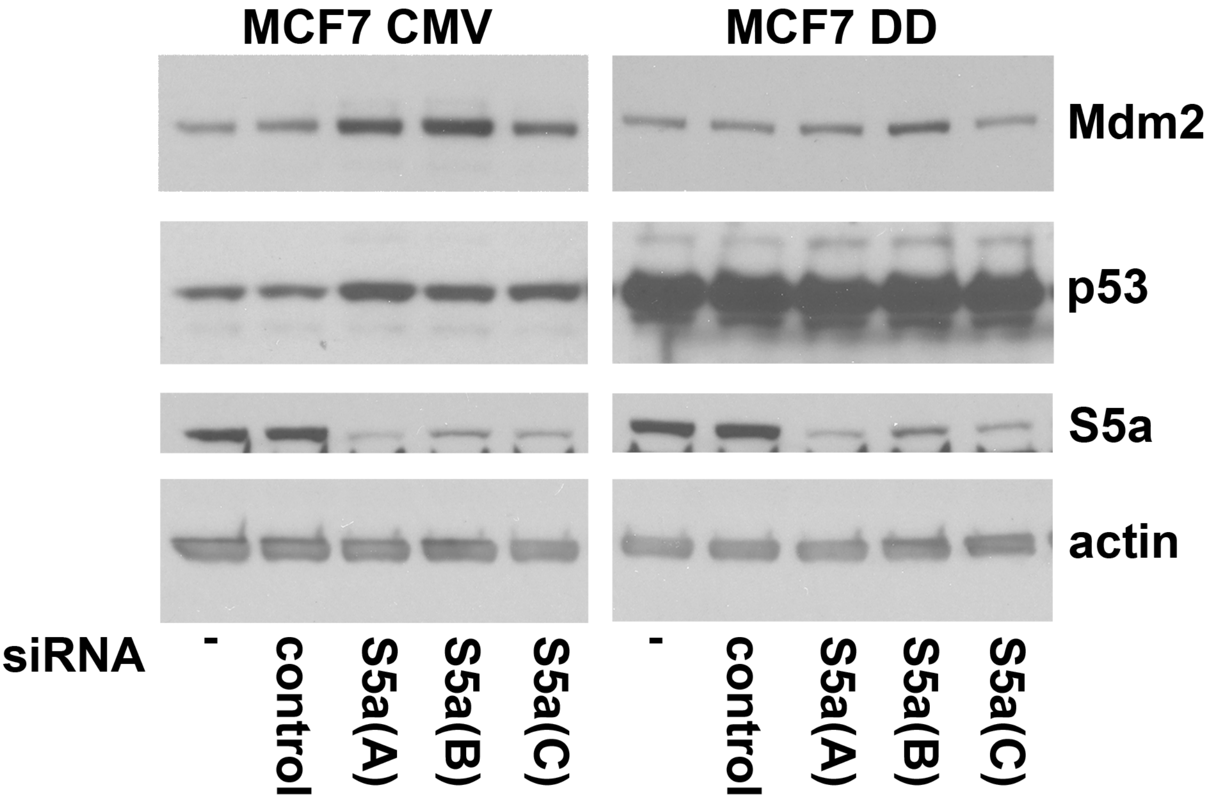

MCF7 CMV
MCF7 DD
Mdm2
p53
S5a
actin
siRNA
-
control
S5a(A)
S5a(B)
S5a(C)
-
control
S5a(A)
S5a(B)
S5a(C)

b

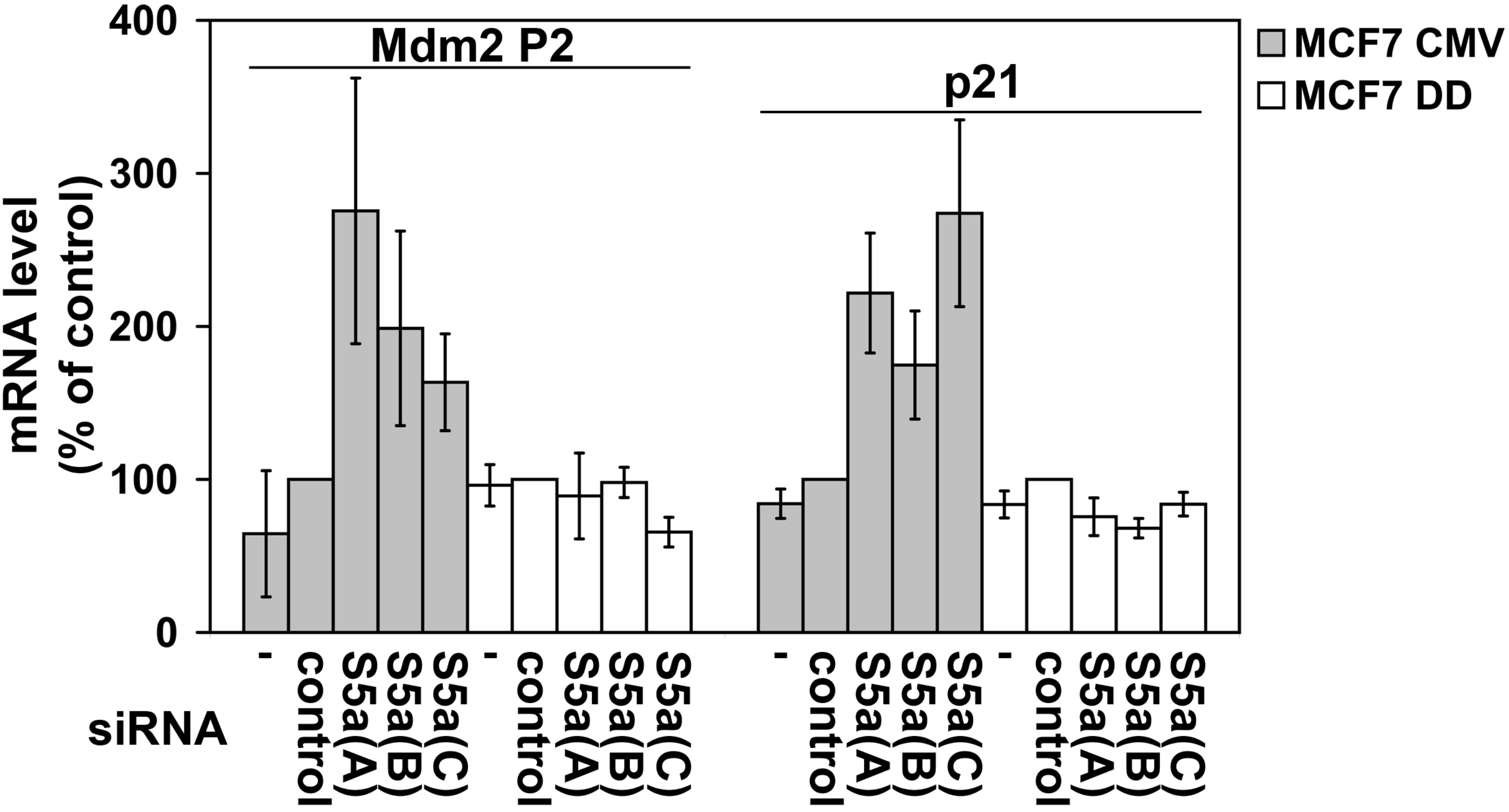

400
300
200
100
0
mRNA level
(% of control)
Mdm2 P2
p21
MCF7 CMV
MCF7 DD
siRNA
-
control
S5a(A)
S5a(B)
S5a(C)
-
control
S5a(A)
S5a(B)
S5a(C)
-
control
S5a(A)
S5a(B)
S5a(C)
-
control
S5a(A)
S5a(B)
S5a(C)

S10

a

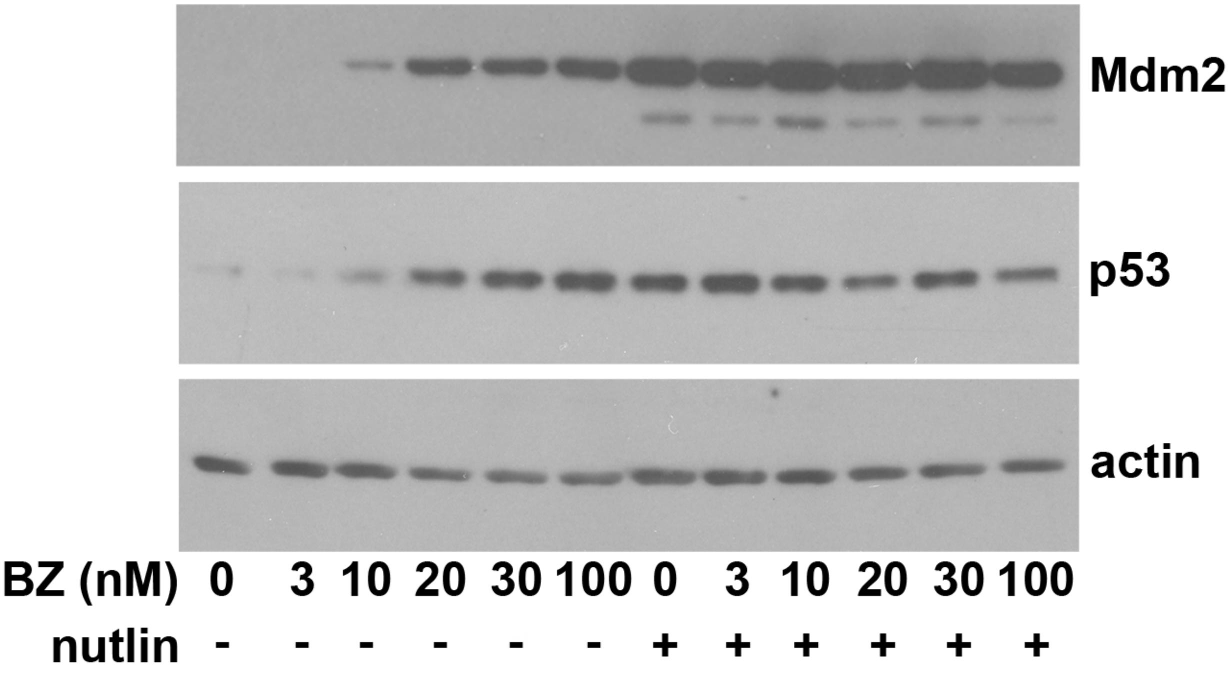
Mdm2
p53
actin
BZ (nM) 0 3 10 20 30 100 0 3 10 20 30 100
nutlin - - - - - - + + + + + +

b

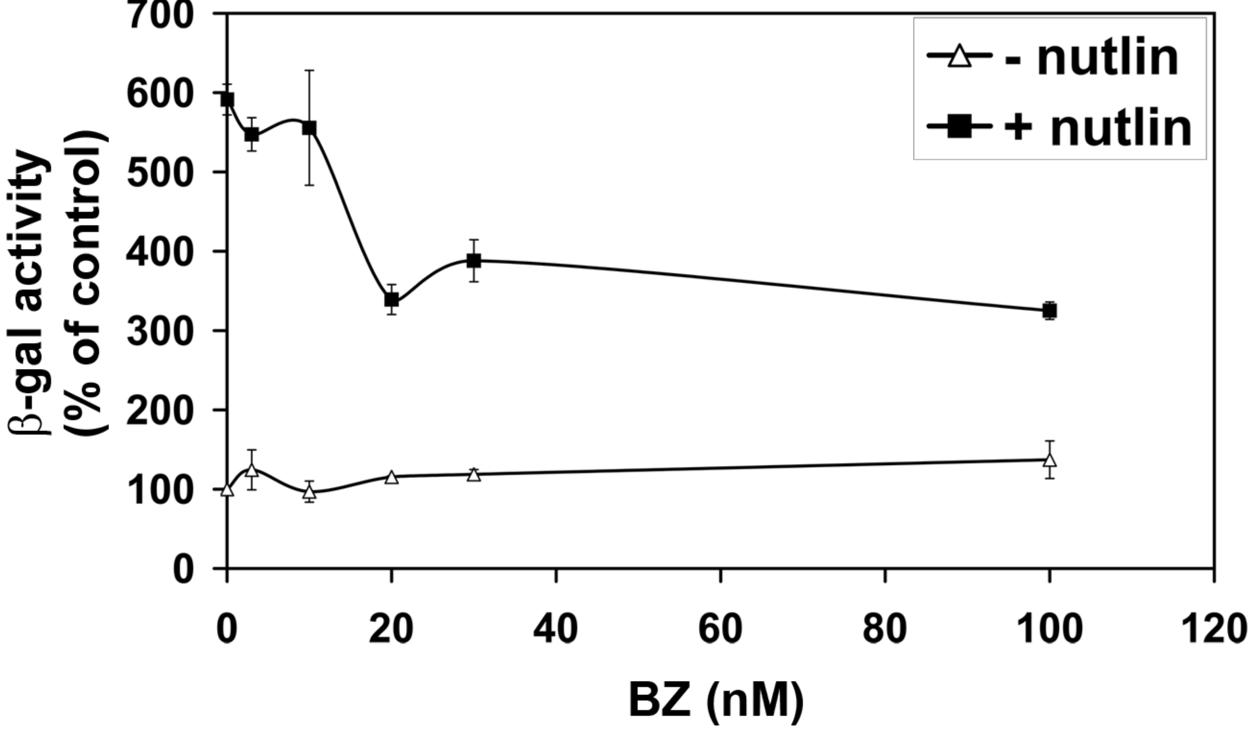
β-gal activity
(% of control)
700
600
500
400
300
200
100
0
0 20 40 60 80 100 120
BZ (nM)
- nutlin
+ nutlin
